# The IL-33-ILC2 axis: a key regulator of type 2 immunity in respiratory disease

**DOI:** 10.3389/fimmu.2026.1891857

**Published:** 2026-07-15

**Authors:** Yue Zhu, Yi Zhang, Rui Cheng

**Affiliations:** 1 Department of Neonatal Medical Center, Children’s Hospital of Nanjing Medical University, Nanjing, Jiangsu, China; 2 Department of Applied Physics, The Hong Kong Polytechnic University, Hong Kong, Hong Kong SAR, China

**Keywords:** asthma, COPD, IL-33-ILC2, pulmonary fibrosis, type 2 immunity

## Abstract

Respiratory diseases represent a major global public health burden, and type 2 immunity plays a key role in their pathogenesis. The interleukin-33 (IL-33)-group 2 innate lymphoid cell (ILC2) axis regulates airway type 2 immune responses, thereby triggering, amplifying, and maintaining pathological inflammation. This review addresses the characteristics of IL-33 and pulmonary ILC2s, including the alarmin function and tissue-specific expression of IL-33, and the origin, localization, functional heterogeneity, and microenvironment-driven plasticity of ILC2s. In particular, we discuss how disease-specific microenvironments shape divergent ILC2 states, including classical type 2 cytokine-producing ILC2s, tissue-repairing AREG-producing ILC2s, ILC1-like cells, and IL-17-producing ILC2-like states. We further review the regulatory mechanisms of the IL-33–ILC2 axis in bronchopulmonary dysplasia, asthma, chronic obstructive pulmonary disease, pulmonary fibrosis, and viral respiratory infections are also discussed, highlighting the remarkable functional plasticity of ILC2s. Targeting the IL-33-ILC2 axis and modulating ILC2 function represent therapeutic strategies for various type 2-mediated airway diseases.

## Introduction

Respiratory diseases such as asthma, chronic obstructive pulmonary disease (COPD), and respiratory viral infections are significant global public health challenges, causing substantial morbidity and mortality. Although the pathophysiological mechanisms underlying these conditions are complex and heterogeneous, the type 2 immune response is known to play a central role in several respiratory diseases. Classically, type 2 immunity combats extracellular parasites and promotes tissue repair. However, abnormal activation of type 2 immunity drives the development of allergic diseases (1, 2). Most studies on type 2 immunity have focused on the role of type 2 helper T (Th2) cells and their specific cytokines (IL-4, IL-5, IL-9, and IL-13) within the adaptive immune response (3). Yet, the initiation of adaptive immunity requires time, failing to fully explain the rapid onset of airway type 2 immunity and the early infiltration of innate immune cells such as eosinophils. However, as the initiation of adaptive immunity takes some time, it cannot explain the rapid onset of type 2 immunity in the airway and the early infiltration of innate immune cells, such as eosinophils in respiratory diseases.

In recent years, progress in research has expanded our knowledge of the innate immune system; notably, the discovery of group 2 innate lymphoid cells (ILC2s), which may be involved in the immediate initiation of type 2 immunity observed in some pathological mechanisms. ILC2s are tissue-resident innate lymphoid cells that do not express antigen-specific receptors but can rapidly produce large quantities of type 2 cytokines upon stimulation by alarmins, thus acting as the vanguard of type 2 immune responses (4, 5). One of the key alarmins in the airway is interleukin-33 (IL-33), which is constitutively expressed within the nuclei of airway epithelial cells, endothelial cells, and fibroblasts. When barrier tissues are compromised by allergens, pathogens, pollutants, or mechanical injury, IL-33 is rapidly released into the extracellular space, serving as a danger signal to activate the immune system (6, 7).

Released IL-33 binds to the high-affinity receptor suppression of tumorigenicity 2 (ST2, also known as IL1RL1) located on the surface of ILC2s, and initiates a potent pro-inflammatory signaling axis (IL-33-ST2-ILC2, hereafter referred to as the IL-33-ILC2 axis). Its activation directly stimulates the proliferation and secretion of IL-5 and IL-13, thereby driving eosinophilic inflammation, mucus hypersecretion, airway hyperresponsiveness, and tissue remodelling—hallmark pathological features of type 2 airway inflammatory diseases (4, 8, 9). Crucially, the IL-33-ILC2 axis functions autonomously and interacts with adaptive immunity; IL-33 directly enhances Th2 cell responses, while ILC2-derived cytokines provide a favorable microenvironment for Th2 cell differentiation and function (6, 10).

This narrative review provides an overview of the IL-33-ILC2 axis as a key regulator of type 2 immunity in airway diseases. We describe the biological characteristics of IL-33 and ILC2s, discuss the activation and regulatory mechanisms of this axis across various airway diseases, and explore the potential of this axis as a target for novel therapeutic strategies. While several excellent reviews have covered the general role of IL-33 and ILC2s in type 2 inflammation, the present review distinguishes itself by placing disease-specific ILC2 functional plasticity and microenvironment-driven phenotypic conversion at the center of the discussion. Specifically, we integrate the concepts of ILC2 heterogeneity (natural vs. inflammatory ILC2s), ILC2-to-ILC1/ILC3-like transdifferentiation, and the dual pathogenic versus tissue-repairing functions of ILC2s to explain how the same IL-33–ILC2 axis can drive divergent disease phenotypes—from eosinophilic asthma and fibrosis to type 1-skewed COPD and post-viral repair. This perspective provides a unifying framework for understanding the context-dependent roles of ILC2s and highlights the therapeutic necessity of precisely modulating, rather than simply blocking, the IL-33–ILC2 axis. This work provides a theoretical foundation for the development of more precise anti-inflammatory therapies.

## IL-33-responsive cells in type 2 immunity

IL-33, a member of the interleukin-1 family, is constitutively expressed in the nuclei of barrier tissue cells such as epithelial, fibroblast, and endothelial cells under homeostatic conditions, while its expression can also be induced in certain immune cells during inflammation (11–16). Upon cellular necrosis or injury, full-length IL-33(IL-33FL) is rapidly released extracellularly and further cleaved by extracellular proteases, such as neutrophil elastase, cathepsin G, and mast cell trypsin, or by the proteases in certain allergens into a mature form with substantially higher ST2-binding affinity and biological activity (17–21). Notably, murine and human IL-33 share only 52% sequence homology and exhibit distinct expression patterns, warranting caution when translating findings from mouse models to human diseases (21–24). Functionally, IL-33 acts primarily as an extracellular alarmin that binds to its receptor ST2, activating NF-κB and MAPK signaling pathways to drive type 2 immune responses (17, 25). Its most critical targets are ILC2s, which respond to IL-33 by rapidly producing IL-5 and IL-13, thereby dominating early allergic airway inflammation (13, 26–28). Furthermore, IL-33 forms a positive feedback loop with Th2 cells and broadly activates other ST2-expressing cells—including Tregs, mast cells, basophils, eosinophils, and dendritic cells—bridging innate and adaptive immunity (29–43).

**Figure 1:**
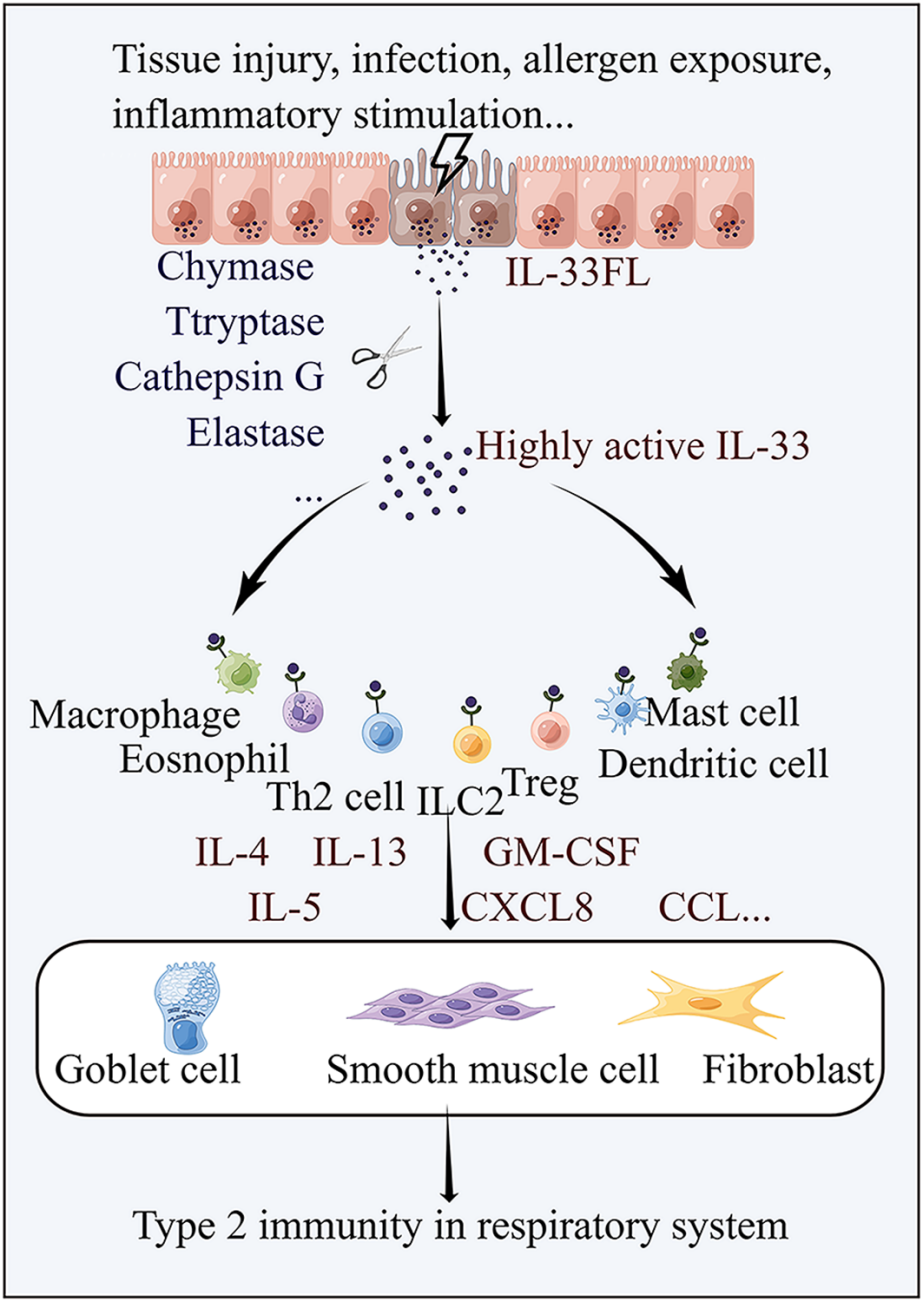
IL-33 is an inducer of type 2 immunity in the respiratory system. Full-length IL-33 (IL-33FL) can be released under various conditions, including tissue injury, necrosis, infection, allergen exposure, pollutants, and inflammatory stimulation. Following release, IL-33FL can be further cleaved by extracellular proteases or proteases inherent to certain allergens, generating a highly active form. Highly active IL-33 stimulates group 2 innate lymphoid cells (ILC2), T helper 2 (Th2) cells, eosinophils, mast cells, and macrophages to produce type 2 cytokines (IL-4 and IL-13) and chemotactic factors. These mediators then act on goblet cells, smooth muscle cells, and fibroblasts to promote mucus production, bronchoconstriction, and airway remodeling—hallmarks of type 2 inflammation in the respiratory tract.

ILC2s, certain regulatory T cells (Tregs), and mast cells are the major tissue-resident cell populations that endogenously express high levels of ST2, thus these cells are the primary targets of IL-33 (44). In contrast, all other cell types that are responsive to extracellular IL-33 either lack ST2 expression in the resting state (and only express ST2 upon activation) or express ST2 in small tissue-specific subpopulations during specific biological processes (21). The differential expression of ST2 (i.e., constitutive vs. inducible) in immune cells under various conditions may account for the reported critical roles of IL-33 in both type 2 and type 1 immune responses and their associated diseases. For example, in mouse models of COPD, ST2 expression on ILC2s is downregulated, whereas ST2 expression on natural killer (NK) cells and macrophages, which is not typically detected at baseline, is upregulated upon activation. Induction of ST2 expression has also been observed on activated Th1 cells and CD8^+^ T cells (24). In lung tissues, IL-33-responsive cells form a multilayered, co-ordinately regulated immunomodulatory network defined by their ST2 receptor expression profiles and localization. Collectively, these cells constitute the core components of the pulmonary type 2 immune response (**Figure 1**). It is important to distinguish between the broader “IL-33-ST2 axis”, which encompasses all ST2-expressing cell types and their diverse functional outputs, and the more specific “IL-33-ILC2 axis”, which refers exclusively to IL-33-mediated activation of ILC2s and the ensuing type 2 cytokine cascade. Although the present review focuses primarily on the IL-33-ILC2 axis as a central driver of type 2 airway pathology, IL-33 can also signal through ILC2-independent pathways—for example, by directly activating CD8^+^ T cells, NK cells, macrophages, or fibroblasts—to elicit type 1 immune responses or non-immune fibrotic effects (24, 45). These ILC2-independent effects are particularly relevant in COPD, viral infections, and pulmonary fibrosis, where they may dominate the pathological outcome, as discussed in the respective disease sections below.

### ILC2

ILC2s constitute the most abundant ST2^+^ cell population in lung tissue and function as the primary effector cells for IL-33 signaling in the lung (24, 46, 47). Activation of ILC2 upregulates ST2 expression in a GATA3- and Gfi1-dependent manner (48, 49). Treatment of Rag2 knockout mice with IL-33 induces production of IL-5 and IL-13; in contrast, no increase in IL-5 or IL-13 production occurs in Rag2 and common γ chain double knockout mice, which retain mast cells and basophils (both of which express ST2 and secrete type 2 cytokines), indicating that this cytokine elevation arises from IL-33-mediated stimulation of ILC2s (50). Further, enhanced IL-33/ST2 signaling has been shown to promote expansion of ILC2s *in vivo* (50, 51).

IL-33 stimulates ILC2s to secrete type 2 cytokines, such as IL-4, IL-5, and IL-13, which in turn recruits eosinophils, mast cells, and Treg cells and drives the polarization of macrophages toward an anti-inflammatory phenotype (M2 activation) (38, 52–54). While this IL-33-activated ILC2 pathway facilitates the clearance of helminth infections, it also underpins the chronic inflammation and fibrosis present in various pathological conditions, including asthma, allergic reactions, insulin resistance and diabetes, obesity, cognitive decline, atopic dermatitis, liver fibrosis, and respiratory fibrosis (37, 55–57). Within the field of respiratory mucosal barrier research, the IL-33-activated ILC2 immune mechanism has been extensively investigated. The mechanism primarily exerts type 2 immune effects that have been implicated in asthma, pulmonary hypersensitivity, microbial lung infections, and lung cancer. This review integrates and analyzes these intrinsic cellular biological correlations, which are elaborated in the subsequent sections.

Notably, the IL-33-ILC2 axis exhibits prominent, double-edged sword immunoregulatory functions. Under specific conditions, such as exposure to cigarette smoke or influenza A virus infection, alveolar epithelial cells release substantial amounts of IL-33 without triggering a typical type 2 inflammatory response: IL-5 and IL-13 levels remain unchanged, and the expression levels of ILC2-associated genes, including *Gata3*, *Il13*, *Il4ra*, *Ccl17*, and *Ccl22*, decrease (24). Further studies revealed that, in the absence of ILC2 involvement, IL-33 can directly activate CD8^+^ T cells and NK cells via the ST2 receptor, promoting interferon-γ production. This initiates a type 1 immune response with antiviral and antitumor effects (24). This functional diversity highlights the pivotal role of the IL-33-ILC2 axis in pulmonary immune regulation.

### Treg cell

A substantial proportion of Tregs constitutively express the ST2 receptor, depending on the tissue type and disease context (21, 38, 58, 59). Studies in colitis, tissue injury models, and graft-versus-host disease (GVHD) have shown that the IL-33/ST2 signaling pathway in Tregs significantly enhances their cellular proportion and immunosuppressive capacity (38, 60). Certain Treg subsets co-express high levels of ST2 and the Th2-associated transcription factor GATA3, and IL-33 maintains their stability and functional integrity (37–39). *In vitro*, IL-33 was shown to promote the proliferation of ST2^+^ Tregs and upregulate GATA3 expression, thereby stabilizing Foxp3 expression and amplifying ST2 expression through a positive feedback loop (38, 39). Interestingly, within the lung microenvironment, CD4^+^Foxp3^+^ Tregs undergo a marked functional transformation upon IL-33 stimulation (37–40). Studies showed that IL-33 not only upregulates GATA3 and ST2 expression in pulmonary Tregs but also induces the production of Th2-type cytokines such as IL-5 and IL-13, leading to a Th2 cell-like phenotype (37–40). Crucially, this conversion is accompanied by a shift in their key functional properties; under the influence of IL-33, pulmonary Tregs lose their inhibitory capacity against effector T cells, transforming from mediators of immunosuppression into potential pro-inflammatory contributors (37–40).Intranasal administration of IL-33 combined with antigen disrupted pre-established antigenic tolerance in the lung (40). In mouse models of IL-33-dependent allergen-driven airway inflammation, lung tissues harbor substantial numbers of dysregulated Foxp3^+^ Tregs with Th2 cell characteristics. This functional deviation of Tregs is particularly prominent in respiratory pathological processes (40). Beyond promoting the conversion of Tregs to Th2-like cells, IL-33 also inhibits Treg differentiation and drives their stable conversion to Th17 cells (61). This multi-directional regulatory pattern of differentiation constitutes a unique immune equilibrium network within the lung tissue.

### Mast cell

Mast cells are characterized by sustained, high-level expression of ST2, a feature that is independent of their tissue origin and maturation/activation status (62, 63). Studies have shown that in both murine and human mast cells in the peritoneal cavity, ST2/IL-33 signaling promotes cell survival by upregulating B-cell lymphoma-X large (Bcl-XL) protein (64). Concurrently, this signaling pathway facilitates mast cell activation and maturation; treatment of CD34^+^ mast cell precursors with IL-33 accelerates their maturation *in vitro* and induces the secretion of granulocyte-macrophage colony-stimulating factor (GM-CSF), IL-5, IL-13, CXCL8, CCL17, CCL22, and CCL2 (65, 66). Extensive evidence indicates that following ST2-mediated signaling, mast cells secrete multiple type 2 cytokines, serving as key mediators of the type 2 immune response (67–69). Activated mast cells also release chymotrypsin and tryptase, two proteases that process IL-33FL into truncated, more potent mature isoforms, thereby amplifying its effects (20). Notably, mast cells also produce IL-33 (15), which, together with the aforementioned proteolytic processing mechanism, forms a self-amplifying positive feedback loop. This comprehensive activation-amplification cascade significantly enhances the intensity and persistence of the pulmonary type 2 immune response.

### Other cell types

In addition to ILC2s, Tregs, and MCs, which endogenously overexpress ST2, various other cell types either only express ST2 within small subpopulations or lack ST2 expression at baseline but can be induced under certain conditions. These include endothelial and epithelial cells, fibroblasts, astrocytes, neurons, and other non-immune cell types (58, 70–74). Importantly, epithelial and endothelial cells and fibroblasts are the major sources of IL-33 production in lung tissue (11, 12, 75). Immune cells that respond to IL-33 in an ST2-dependent manner (excluding MCs, Tregs, and ILC2s) comprise innate immune cells, such as eosinophils, basophils, neutrophils, myeloid-derived suppressor cells (MDSCs), macrophages (Mφ), NK cells, and dendritic cells (DCs), and adaptive immune cells, such as natural killer T (NKT) cells, CD4^+^ T cell subsets (Th1/2/17), CD8^+^ T cells, and B cells (76–82).

Of these, the IL-33 response mechanism in Th2 cells merits particular attention. ST2 was initially identified as a marker expressed by highly differentiated Th2-type CD4^+^ T cells (83). Naïve CD4^+^ T cells express low levels of ST2; however, upon activation, ST2 expression is significantly upregulated, giving these cells heightened sensitivity to IL-33 (84, 85). Findings from mouse models support the role of IL-33 in mediating Th2 CD4^+^ T cell differentiation and activation (35). Functionally, IL-33 enhances cytokine secretion by Th2 cells, as evidenced by the markedly higher levels of IL-5 and IL-13 produced by antigen-specific ST2^+^ Th2 cells than that produced by ST2^-^ Th2 cells, and exhibits chemotactic activity, directing adoptively transferred Th2 cells to IL-33-enriched sites *in vivo* (17, 86). However, ST2 is not explicitly required for the Th2 response; in nematode infection and asthma models, Th2 cell-mediated immune responses and recruitment to inflammatory sites can occur independently of ST2 signaling, indicating functional redundancy or alternative pathways (87). Concurrently, Th2 cells and ILC2s form a finely regulated synergistic network during immune responses, ensuring effective initiation and sustained amplification of type 2 immunity (29) (**Table 1**).

**Table 1:** IL-33-responsive cell types: ST2 expression, function, and evidence.

Cell type	ST2 expression pattern	Main function	Refs
ILC2s	Constitutively high; further upregulated by GATA3 and Gfi1 upon activation	Primary IL-33 targets in the lung; rapid production of IL-5, IL-13; drive type 2 inflammation and tissue repair	(13, 25, 50)
Tregs	Constitutively expressed; tissue- and context-dependent	Enhanced proliferation, suppressive function, and GATA3/Foxp3 stability; in the lung, can lose suppressive capacity and acquire a Th2-like phenotype under IL-33	(38–40)
Mast cells	Constitutively high (independent of tissue origin and maturation/activation status)	Survival (Bcl-XL), maturation; secretion of type 2 cytokines (IL-5, IL-13, etc.) and proteases (tryptase, chymase) that cleave IL-33FL into more active forms; also produce IL-33, forming a self-amplifying loop	(15, 20, 65)
Th2 cells	Inducible upon activation; ST2 is a marker of highly differentiated Th2 cells	Enhanced IL-5 and IL-13 secretion; chemotaxis toward IL-33	(17, 83)
CD8^+^ T cells	Inducible (e.g., by cigarette smoke or viral infection)	Production of IFN-γ; contribution to type 1 antiviral and antitumor immunity	(24)
NK cells	Inducible (e.g., by cigarette smoke, IL-12)	Production of IFN-γ; antiviral and antitumor responses	(24, 78)
Macrophages	Inducible (e.g., in fibrosis and COPD)	M2 polarization, TGF-β production, contribution to fibrosis	(45)
Fibroblasts	Inducible (e.g., in fibrotic niches)	TGF-β and extracellular matrix production; promotion of fibrosis	(45)
Eosinophils	Inducible	Maturation, survival, and cytokine production	(81)
Basophils	Inducible	IL-4 production that enhances ILC2 activation	(154)
Dendritic cells	Inducible	Promotion of Th2 differentiation	(16)
Th1 cells	Inducible upon activation	Production of IFN-γ; involvement in type 1 responses	(34)
B cells	Inducible	Production of regulatory molecules (e.g., PIBF1); protection in certain contexts	(76)
Epithelial cells	Low/inducible (also major source of IL-33)	Production of inflammatory mediators (e.g., IL-17F); possible involvement in barrier repair	(11, 41, 43)
Endothelial cells	Low/inducible (also source of IL-33)	Activation, angiogenesis, vascular remodeling	(11, 71)

## Characteristics of pulmonary ILC2

### Origin and development of ILC2

Differentiation of ILC2s is tightly regulated by a multi-layered transcriptional network. ILC2s originate from hematopoietic stem cells (HSCs), and their differentiation proceeds through common lymphoid progenitors (CLPs), followed by a series of key progenitor cell subsets before maturing into ILC2s. Specifically, HSCs differentiate into CLPs (88, 89), which can either give rise to lymphocytes or further differentiate into either early innate lymphoid progenitors (EILPs) or common innate lymphoid progenitors (CILPs). The transition from CLPs to EILPs may be driven by Notch signaling, which induces the expression of the transcription factors Tox, NFIL3, and TCF-1 (90–92). Notably, the lack of Notch receptors on CILPs suggests that attenuated Notch signaling may facilitate their differentiation from EILPs (92). Subsequently, CILPs differentiate into common helper innate lymphoid progenitors (CHILPs), followed by innate lymphoid progenitor cells (ILCPs), and ultimately ILC2Ps. CHILPs depend on the transcription factor ID2, a key regulator of the development of all mature ILC subsets (93). The expression level of GATA3 also plays a critical role in the fate determination of CHILPs toward ILCPs; CHILPs with lower GATA3 expression differentiate into lymphoid tissue-inducing cells, whereas those with higher GATA3 expression tend toward the ILCP lineage (94). Within the ILCP population, ILC2Ps are the direct precursors of mature lung-resident ILC2s. Ultimately, ILC2Ps differentiate into mature ILC2s under the combined regulatory action of multiple transcription factors, including RORα, GATA3, GFI1, ETS1, and BCL11B (4, 95).

### Localization and migration of pulmonary ILC2

Tissue-resident ILC2s colonize the lungs during the perinatal and neonatal periods and constitute the primary resident ILC subset in the lung (96). This early colonization is dependent on the IL-33 signaling pathway (97), and developing ILC2s already possess the capacity to produce IL-5 and IL-13 upon entering lung tissue. These tissue-resident ILC2s are permanent residents, maintaining population stability through the acquisition of tissue-specific identity and self-renewal mechanisms (98, 99). Studies have shown that pulmonary ILC2s are predominantly localized to the perivascular niche within medium-to-large airways (100), forming close spatial associations with IL-33-producing mesenchymal fibroblasts. This distinct microenvironmental architecture is considered pivotal for shaping the specific phenotypic and functional properties of pulmonary ILC2s (99, 101).

In addition to self-renewal, maintenance of pulmonary ILC2 populations also relies on replenishment from circulating progenitor cells, bone marrow precursors, and mature ILC2s migrating from other tissues (102–104). Experimental induction of allergic pulmonary inflammation reduced the frequency of ILC2s within the bone marrow, followed by an increase in pulmonary ILC2 numbers—strongly suggesting directed migration of ILC2s from the bone marrow to inflamed tissue (105). In this context, IL-33 not only functions as a key activator of ILC2s in peripheral organs (106), but also regulates inflammation-induced extramedullary ILC2 hematopoiesis and subsequent hematogenous migration to the lungs (103). Indeed, following intranasal administration of recombinant IL-33, ILC2s accumulated in the peribronchial and perivascular spaces of mouse lungs, which was dependent on CCL8-CCR8 ligand-receptor interaction (106). Studies have reported upregulation of the chemokine receptor CCR8 in lung-infiltrating ILC2s following IL-33 exposure, which was accompanied by elevated pulmonary levels of its cognate ligand CCL8. Functional evidence indicates that antibody-mediated CCR8 blockade significantly suppresses inflammation-induced ILC2 accumulation in peribronchial tissue *in vivo* (106). Beyond chemokine signaling, the presence of perivascular adventitial cells has been shown to significantly promote the spatial aggregation of pulmonary ILC2s around pulmonary arteries under inflammatory conditions (100, 107). Bidirectional adventitial matrix cell-ILC2 interactions—mediated by IL-33 and thymic stromal lymphopoietin (TSLP) derived from adventitial matrix cells and IL-13 derived from ILC2s—are triggered by inflammatory stimuli and generate an optimized microenvironment that enhances ILC2 enrichment and activation (100). Notably, both IL-33 stimulation and helminth infection induce ILC2 migration from the lungs into the circulatory system, a process involving complex signaling pathways, including those mediated by sphingosine-1-phosphate receptors and tryptophan hydroxylase 1 (108–111).

Recent studies have further elucidated the functional heterogeneity of pulmonary ILC2s, which primarily comprise two distinct functional subpopulations: natural ILC2s (nILC2s) and inflammatory ILC2s (iILC2s) (112). As a tissue-resident population, nILC2s express high levels of the IL-33 receptor ST2 and primarily secrete IL-5 and IL-13. In contrast, iILC2s, as a migratory population, express high levels of the IL-25 receptor IL-17RB, and, in addition to IL-5 and IL-13, can also produce IL-4 and IL-17A (102). These two subpopulations exhibit marked differences in transcription factor dependency, tissue distribution, and functional characteristics. Studies indicate a polygenic origin for the iILC2s in lung tissue from multiple tissues, including the gut, bone marrow, and the lung itself (111). Although evidence for an intestinal origin is relatively robust (e.g., shared expression of α4β7, CCR9, and the alarmin receptor IL-17RB) (102, 111, 112), lung-derived iILC2s exhibit unique gene expression profiles, namely, the absence of arginase 1 and expression of IL-4/IL-17 (50, 102, 109, 112–115), suggesting significant phenotypic reprogramming during migration. Furthermore, the post-activation migratory capacity of resident pulmonary ILC2s (106, 108, 109) and contributions from bone marrow progenitor cells complicate their origin (93, 103, 104, 116). More precise lineage-tracing techniques are required to elucidate the specific contributions of each tissue to the formation of the lung iILC2 population. Building upon our current understanding of the development, localization, and functional heterogeneity of ILC2s within lung tissue, we will next focus on the core mechanisms of the IL-33-ILC2 axis in respiratory diseases.

### Functional heterogeneity and microenvironment-driven plasticity of ILC2s

The concept that ILC2s are a uniform population of type 2 cytokine-producing cells has been substantially revised by the recognition of their remarkable functional heterogeneity and plasticity. This heterogeneity operates at two levels: pre-existing, developmentally programmed subsets, and inducible, microenvironment-driven functional states that can cross traditional ILC subset boundaries.

#### Developmentally defined heterogeneity: nILC2s versus iILC2s

In mouse models, pulmonary ILC2s comprise at least two developmentally and functionally distinct subsets: natural ILC2s (nILC2s) and inflammatory ILC2s (iILC2s) (102, 112). nILC2s are tissue-resident cells that express high levels of ST2 and respond predominantly to IL-33 by producing IL-5 and IL-13. In contrast, iILC2s are a more migratory population expressing high levels of the IL-25 receptor IL-17RB and, in addition to IL-5 and IL-13, can produce IL-4 and IL-17A (102). These subsets also differ in their transcription factor dependencies and tissue distribution. Although the nILC2/iILC2 dichotomy is well characterized in mice, the extent to which analogous subsets exist in the human lung remains an active area of investigation, with single-cell RNA sequencing studies beginning to reveal similar functional diversity in human pulmonary ILC2s.

#### Microenvironment-driven plasticity: conversion to ILC1- or ILC3-like phenotypes

Beyond developmentally programmed heterogeneity, ILC2s exhibit a remarkable capacity for microenvironment-driven phenotypic conversion. In mouse models and *in vitro* human ILC2 cultures, exposure to specific cytokine milieus can drive ILC2s to acquire features of other ILC subsets in a process often termed transdifferentiation or plasticity. IL-12 and IL-18, two cytokines prominently elevated in cigarette smoke-exposed and chronically inflamed lungs, can downregulate GATA3 and ST2 expression while upregulating T-bet and IFN-γ production, effectively converting ILC2s into ILC1-like cells (24, 117, 118). Similarly, IL-1β in combination with IL-18 can induce ILC2s to produce IL-17A, adopting an ILC3-like phenotype (119). This plasticity is not merely an *in vitro* curiosity; in mouse models of COPD, viral infection, and allergic inflammation, adoptively transferred ILC2s can give rise to IFN-γ-producing or IL-17A-producing progeny *in vivo* (118). In human respiratory diseases, direct *in vivo* evidence for such conversion remains limited. The recent identification of IL-17A^+^ ILC2s in sputum from severe asthma patients (119) and the inverse correlation between ILC2 and ILC1 frequencies in COPD lungs provide suggestive but not definitive evidence for ILC2 plasticity in human disease (24, 120).

#### Stable subsets versus dynamic adaptation

A fundamental unresolved question is whether the distinct ILC2 functional states represent stable, lineage-committed subsets or dynamic, reversible adaptations to the local cytokine environment. Mouse lineage-tracing and adoptive transfer experiments strongly favor the latter model: ILC2s that have acquired ILC1-like features in an IL-12/IL-18-rich environment can revert to a type 2 phenotype upon removal of those polarizing signals (117, 118), indicating that their functional identity is continuously shaped by the microenvironment rather than fixed by irreversible differentiation. Whether the same dynamic regulation occurs in human tissue-resident ILC2s remains to be determined. This distinction carries important therapeutic implications: if ILC2 functional states are reversible, then therapies that target the polarizing cytokine milieu (e.g., anti-IL-12, anti-IL-1β) could restore protective ILC2 functions; if they represent stable, pathogenic subsets, selective depletion strategies may be required.

#### Common microenvironmental drivers

The functional state of ILC2s is dictated by the integrated input of multiple microenvironmental signals. IL-33 promotes ILC2 proliferation and canonical type 2 cytokine production. TSLP, often co-released with IL-33 from damaged epithelium, enhances ILC2 survival and induces steroid resistance (121). IL-25 preferentially expands iILC2-like populations and synergizes with IL-33 to amplify type 2 responses (102, 122). IL-12, IL-18, and IL-1β act as the principal drivers of ILC2 plasticity toward ILC1- or ILC3-like phenotypes (117, 118). Type I and type II interferons, conversely, suppress ILC2 function and can restrain both canonical type 2 cytokine production and aberrant plasticity (123, 124). The relative abundance of these signals, which varies substantially across different respiratory diseases and disease phases, determines whether ILC2s function as canonical type 2 effectors, convert into ILC1/ILC3-like pro-inflammatory cells, or assume tissue-repairing functions through AREG and LIF production.

#### Relevance to respiratory disease

This integrated view of ILC2 heterogeneity and plasticity provides a mechanistic framework for understanding the divergent roles of the IL-33-ILC2 axis across respiratory diseases. In eosinophilic asthma, a microenvironment rich in IL-33 and TSLP favors canonical type 2 ILC2 function (119, 125, 126), whereas in severe asthma, elevated IL-1β and IL-18 may additionally drive IL-17-producing ILC2s that contribute to mixed granulocytic inflammation (117, 119). In COPD, the cigarette smoke-induced IL-12/IL-18-rich milieu promotes ILC2-to-ILC1 conversion, shifting the axis toward type 1 inflammation (24, 118), a process that is reversed during virus-induced exacerbations when a surge of IL-33 transiently restores ILC2 dominance (127). In pulmonary fibrosis, the interplay between IL-33, TGF-β, and AREG shapes whether ILC2s promote tissue repair or pathological collagen deposition (128, 129). During viral infections, the timing and magnitude of the interferon response critically dictate whether ILC2s drive immunopathology or facilitate epithelial repair (123, 124). This disease-specific, plasticity-centered perspective is a central theme of this review and is elaborated in detail in the following sections.

## IL-33 and ILC2 in respiratory disease

In the following sections, we review the current understanding of IL-33, ST2, and ILC2s in the context of specific respiratory diseases, including asthma, COPD, bronchopulmonary dysplasia (BPD), and pulmonary fibrosis. This review is limited to these disorders, and lung cancer is excluded from the discussion (**Table 2**).

**Table 2:** Summary of the IL-33/ILC2 axis across respiratory diseases.

Disease/Subtype	Source of IL-33	ILC2 phenotype/subset	Key downstream cytokines/mediators	Type of evidence	Major pathological outcomes	Refs
Lung Development	Alveolar epithelial cells and stromal cells stimulated by the first breath	Tissue-repairing ILC2 (AREG^+^) and pro-inflammatory ILC2 (IL-5^+^IL-13^+^)	IL-5, IL-13, AREG	Mouse models (IL-33 KO does not impair alveolarization)	Establishes the type 2 immune milieu; supports alveolarization (non-essential)	(96, 97, 133–136)
Bronchopulmonary Dysplasia (BPD)	Hyperoxia-induced release from stressed/necrotic lung epithelial and stromal cells	Overactivated pro-inflammatory ILC2 (IL-13^+^, Arg1^+^, Klrg1^+^)	IL-13 (major), IL-5	Hyperoxia-exposed neonatal mouse models; human infant serum	Arrested alveolarization, pulmonary inflammation, airway hyperresponsiveness	(136–141)
Asthma – Type 2-high	Allergen/virus-damaged airway epithelium	Classical ST2^+^ ILC2 (IL-5^+^IL-13^+^)	IL-5, IL-13	Genetic association studies; murine allergen models; human BAL/airway tissue	Eosinophilic infiltration, mucus hypersecretion, airway hyperresponsiveness	(125, 126, 143–149)
Asthma – Mixed Granulocytic	Damaged epithelium (IL-33 release)	HMGB1-secreting ILC2; IL-17^+^ ILC2 (transdifferentiated)	HMGB1, IL-17	Mouse models; analysis of human sputum ILC2s	Mixed eosinophilic-neutrophilic airway infiltration	(119, 150)
Asthma – Steroid Resistance	Epithelium-released alarmins (IL-33, TSLP, TL1A)	TSLP-induced steroid-resistant ILC2; IL-9^+^ ILC2 (induced by IL-33+TL1A)	IL-9, IL-5, IL-13	Mouse models; *in vitro* assays; observations in severe human asthma	Persistent type 2 inflammation refractory to corticosteroid treatment	(121, 151–154)
COPD – Current Smokers	Cigarette smoke-exposed airway epithelium	ILC2-to-ILC1 plasticity (IFN-γ^+^ ILC1-like cells); ILC2 numbers decreased	IFN-γ (from converted ILC1-like cells); IL-5/IL-13 during exacerbations	Mouse smoke models; human lung tissue and blood analysis	Type 1-dominated chronic inflammation, emphysema	(24, 95, 118, 120, 158, 160, 164, 171)
COPD – Former Smokers	Residual epithelial stress/alarmin release	Partially restored ILC2 function; reduced ILC1 conversion	IL-5, IL-13 (responsive to anti-IL-33 therapy)	Phase 2a clinical trial (itepekimab)	Responsive to IL-33 blockade with reduced exacerbations	(161)
COPD – Eosinophilic COPD	Airway epithelium (similar to asthma triggers)	Activated ILC2 (ST2^+^, Arg1^+^); increased cell numbers	IL-5, IL-13	Clinical trials (dupilumab); observational cohort studies	Eosinophilic airway inflammation, exacerbations; responsive to type 2 biologics	(159, 160, 165–168)
COPD – Asthma-COPD Overlap (ACO)	Mixed asthma/COPD triggers	Heterogeneous ILC2/ILC1 features	IL-5, IL-13, and potentially IFN-γ	Clinical and molecular profiling studies	Mixed eosinophilic and neutrophilic inflammation; variable steroid response	(117, 127, 164)
Pulmonary Fibrosis – Bleomycin Model	Alveolar epithelial cells and macrophages post-injury	IL-13-producing ILC2	IL-13 (key fibrotic effector), AREG, TGF-β amplification	Mouse model with adoptive transfer and depletion experiments	IL-13-dependent collagen deposition and fibrosis	(45, 128, 173, 174)
Pulmonary Fibrosis – IPF	Persistent epithelial injury and aberrant repair	Increased ILC2 numbers (Regnase-1 low); Nrp1/ST2 upregulation	IL-13, AREG, TGF-β	Human BALF/tissue analysis; mouse models targeting Nrp1/ST2	Progressive fibroblast activation and collagen deposition; poor prognosis	(45, 173, 175–178)
Pulmonary Fibrosis – CF	CFTR dysfunction, chronic epithelial stress and infection	Expanded ILC2 driven by IL-9/mast cell loop	IL-13, IL-9	CF patient samples and mouse models	Airway remodeling, bronchiectasis, mucus obstruction	(172, 181)
Pulmonary Fibrosis – SSc-ILD	Epithelial/endothelial injury in autoimmune context	Increased ILC2 in blood/lung (direct functional evidence limited)	IL-13, TGF-β	Clinical correlation studies (serum IL-33/IL-13); preclinical models	Skin and lung fibrosis; part of a systemic autoimmune-fibrotic process	(174, 185)
Viral Infection – Influenza	NLRP3 inflammasome in alveolar macrophages and NKT cells	Dual: early pathogenic (IL-5/IL-13^+^) to late protective (AREG^+^ LIF^+^)	IL-5, IL-13 (early); AREG, LIF (late)	Mouse infection models; BATF-dependent regulation	Early immunopathology (AHR, eosinophilia) followed by tissue repair	(123, 182, 187–189)
Viral Infection – RSV	Epithelial cells, with TSLP playing a dominant role	Sustained pathogenic ILC2 activation	IL-13, IL-5	Neonatal mouse models; severe infant bronchiolitis studies	AHR, mucus hypersecretion, eosinophilia; increased risk of long-term asthma	(190–198)
Viral Infection – RV	Epithelial cells; IL-25 is critical initiator, IL-33/TSLP synergistic	IL-13-producing ILC2	IL-13	Neonatal mouse models; RORα blockade is protective	Mucous metaplasia, airway hyperresponsiveness	(183, 199–202)
Viral Infection – SARS-CoV-2	Not well defined (likely epithelial); regulated by IFN milieu	Dynamic: suppressed by high IFN-γ; putative protective AREG^+^ ILC2 when IFN signaling wanes	AREG (putative in repair); IL-5/IL-13 not clearly defined	Observational studies in COVID-19 patients; parallels with influenza models	Variable; outcomes linked to IFN dysregulation and cytokine microenvironment	(182, 188, 203, 204)

### Lung development and BPD

#### Physiological role in normal lung development

Lung development is a complex process involving intricate crosstalk among multiple cell types. The most active phase of lung development, namely alveolar septation, occurs during the second postnatal week in mice and extends to 2–3 years of age in humans. This developmental timeline coincides with the establishment of a type 2 immune microenvironment in the lung (97, 130, 131), wherein the IL-33-ILC2 axis plays a dual role: it contributes to the construction of an early type 2 immune milieu that supports normal development, while also harboring the potential to trigger pathological risks under adverse conditions.

The colonization and expansion of ILC2s in the neonatal lung represent a physiological process intimately linked to development. ILC2s colonize the lungs during embryogenesis (mid-gestation in humans, embryonic day 17.5 in mice) and undergo rapid proliferation in the early postnatal period: a time window that closely overlaps with the critical stage of alveolarization (96, 98, 99, 132).Under physiological conditions, the mechanical and oxidative stresses associated with the first postnatal breath induce alveolar epithelial cells and stromal cells in the outer membrane to produce IL-33, which subsequently activates ILC2s via binding to its cognate receptor ST2, thereby promoting proliferation of ILC2s and the secretion of type 2 cytokines, including IL-5 and IL-13 (96, 133–135). In mouse models, this early-life IL-33-ILC2 activation enhances Th2 responses and facilitates the establishment of a pulmonary type 2 immune microenvironment (97). However, studies using IL-33-deficient mice have shown that IL-33 deficiency does not have overt detrimental effects on alveolarization (134). This observation suggests that early-life IL-33 signaling may be more critical for the induction of ILC2-driven type 2 immunity, whereas lung development may proceed via alternative or compensatory pathways.

#### Pathological activation in BPD

In stark contrast to its physiological supportive role during normal development, the IL-33-ILC2 axis exhibits significant pathological dysregulation in BPD. Notably, neonatal pulmonary ILC2s exhibit pronounced functional heterogeneity. Using a ROR-α lineage-tracing model, two distinct ILC2 subpopulations with divergent functional properties have been identified in the neonatal mouse lung: a pro-inflammatory subset that secretes Th2 cytokines, and a tissue-repairing subset that expresses amphiregulin (AREG) (136). Among these, the pro-inflammatory subset is strictly dependent on IL-33 signaling (136, 137).

**Figure 2:**
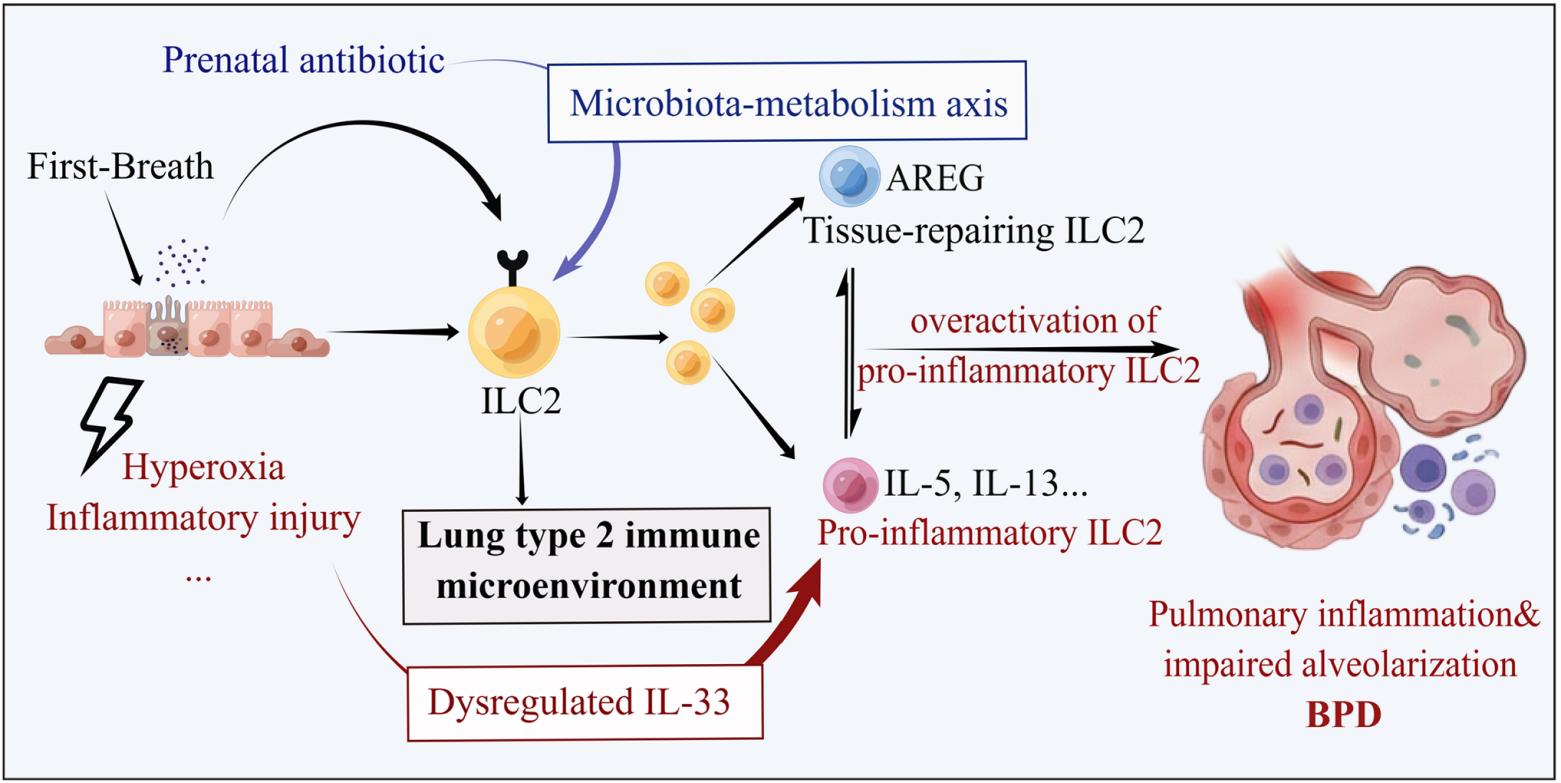
Regulation of lung development and BPD by the IL-33-ILC2 axis. Mechanical and oxidative stress from the first postnatal breath induces IL-33 release from lung epithelial cells, which in turn activates ILC2s. ILC2s differentiate into a pro-inflammatory subset (secreting IL-5 and IL-13) and a tissue-repairing subset (secreting AREG). The IL-33-ILC2 axis contributes to the construction of an early type 2 immune microenvironment that supports normal lung development under physiological conditions, though it is not indispensable; under pathological conditions such as BPD, dysregulation of this axis (particularly overactivation of pro-inflammatory ILC2s) plays a central role in disease pathogenesis. Furthermore, prenatal antibiotic exposure can modulate neonatal pulmonary ILC2 numbers by altering the microbiota–metabolism axis.

Hyperoxia, inflammatory injury, and other major drivers of BPD can induce excessive cellular stress and necrosis in the developing lung, leading to massive, uncontrolled IL-33 release. This phenomenon has been validated both in hyperoxia-exposed neonatal mouse models of BPD and in peripheral blood samples from human infants with BPD (138, 139). Such aberrant IL-33 surges disrupt the functional balance and heterogeneity of neonatal ILC2s, resulting in excessive activation of specific pro-inflammatory ILC2 subsets. In animal model evidence, overactivation of these pro-inflammatory ILC2 subsets leads to IL-13 overproduction, which impairs alveolar formation and exacerbates pulmonary inflammation (137, 140). Blockade of type 2 cytokines attenuates these effects and improves lung function in mouse models of BPD (141). Furthermore, in mouse models, environmental factors, such as prenatal antibiotic exposure, can modulate neonatal pulmonary ILC2 numbers by altering the microbiota-metabolism axis (e.g., via regulation of butyrate production), which in turn correlates closely with the development of long-term airway hyperresponsiveness (142). In summary, the IL-33-ILC2 axis contributes to the construction of an early type 2 immune microenvironment that supports normal lung development under physiological conditions, though it is not indispensable; however, under pathological conditions such as BPD, dysregulation of this axis (particularly overactivation of pro-inflammatory ILC2s) plays a central role in disease pathogenesis (**Figure 2**). In terms of translational implications, these findings suggest that strategies aimed at dampening pro-inflammatory ILC2 activity while preserving tissue-repairing ILC2 functions may offer a novel therapeutic approach for BPD; however, the absence of direct *in vitro* studies using human neonatal ILC2s represents a gap that needs to be addressed before clinical translation.

### Asthma

Asthma is a chronic allergic respiratory disease characterized by persistent airway inflammation and variable airflow obstruction. The inflammatory process is broadly classified into type 2-high and type 2-low phenotypes, with the majority of patients presenting with the type 2-high phenotype driven by Th2 cells and ILC2s. Within this framework, the IL-33-ILC2 axis plays a multifaceted role that extends beyond classical type 2 immunity to encompass mixed granulocytic inflammation and steroid resistance, both of which are hallmarks of severe, difficult-to-treat asthma.

#### The IL-33-ILC2 axis as a core driver of type 2-high asthma

Human genetic studies have established that polymorphisms in the *IL-33*, *ST2*, *RORα*, and *IL-13* genes are strongly associated with the type 2-high asthma phenotype, as these genes play critical roles in the development and functional activation of ILC2s (143). Recent epigenomic analyses have further shown that regulatory elements adjacent to these genes in ILC2s are significantly linked to asthma susceptibility (144). Mechanistically, the axis is rapidly activated upon disruption of the airway epithelial barrier by allergens or viruses, leading to the release of IL-33 and its proteolytic processing into a highly active mature form. IL-33 directly activates resident pulmonary ILC2s via ST2, driving their proliferation and robust secretion of IL-5 and IL-13, which in turn orchestrate eosinophil infiltration, mucus hypersecretion, and airway hyperresponsiveness. Murine studies confirm this pathway: pulmonary IL-33 levels rise within hours of allergen exposure and remain elevated during chronic inflammation, while mice deficient in *Il1rl1* or *Tslpr* exhibit markedly attenuated allergic airway inflammation (125, 145). Crucially, ILC2s are the principal IL-33-responsive cells mediating these features; intranasal IL-33 challenge fails to induce significant type 2 cytokine production or eosinophilia in ILC2-deficient mice (146). In patients with asthma, the levels of IL-33 and ILC2s in the airways and circulation are positively correlated with disease severity and mirror the expression patterns of IL-5 and IL-13 (126, 147–149).

#### ILC2 plasticity and mixed granulocytic inflammation

Beyond driving pure eosinophilic inflammation, the IL-33-ILC2 axis contributes to mixed granulocytic asthma, a phenotype frequently observed in severe disease. Two principal mechanisms have been identified. First, activated ILC2s secrete high-mobility group box 1 (HMGB1), a potent chemoattractant and activator of neutrophils, thereby recruiting neutrophils into the airway and fostering a mixed eosinophilic-neutrophilic infiltrate (150). Second, recent human sputum data identified c-kit^+^IL-17A^+^ ILC2-like cells in individuals with severe asthma, suggesting that ILC2s may acquire ILC3-like inflammatory features under specific microenvironmental conditions (119). These IL-17-producing ILC2-like states may promote neutrophilic inflammation while retaining elements of type 2 cytokine production, thereby providing a potential explanation for the coexistence of eosinophilic and neutrophilic inflammation in severe asthma. However, this interpretation should remain cautious. Current human evidence supports phenotypic heterogeneity and plasticity-associated signatures, but does not yet definitively establish stable or irreversible ILC2-to-ILC3 transdifferentiation. Larger cohorts and lineage-resolved functional analyses are needed to determine whether these cells represent a stable subset, a transient activation state, or a dynamic adaptation to severe airway inflammation.

#### Steroid resistance in the IL-33-ILC2 axis

A critical barrier to the effective treatment of severe asthma is corticosteroid resistance, to which the IL-33-ILC2 axis contributes directly. In mouse models, the alarmin TSLP, which is co-released with IL-33 from injured airway epithelium, induces corticosteroid resistance in ILC2s, enabling them to persist and sustain type 2 cytokine production despite steroid treatment (121). *In vitro* mechanistic evidence demonstrates that TNF-like protein 1A (TL1A) synergizes with IL-33 to drive the differentiation of IL-9-producing ILC2s via STAT5 activation, and these IL-9^+^ ILC2s are relatively resistant to corticosteroid-induced apoptosis (151–153). In mouse studies, basophil-derived IL-4, induced by IL-33, further enhances ILC2 activation in a positive feedback loop that may operate independently of steroid-sensitive pathways (154). Together, these mechanisms implicate the broader alarmin-ILC2 network, orchestrated by IL-33, as a central mediator of steroid-refractory airway inflammation.

**Figure 3:**
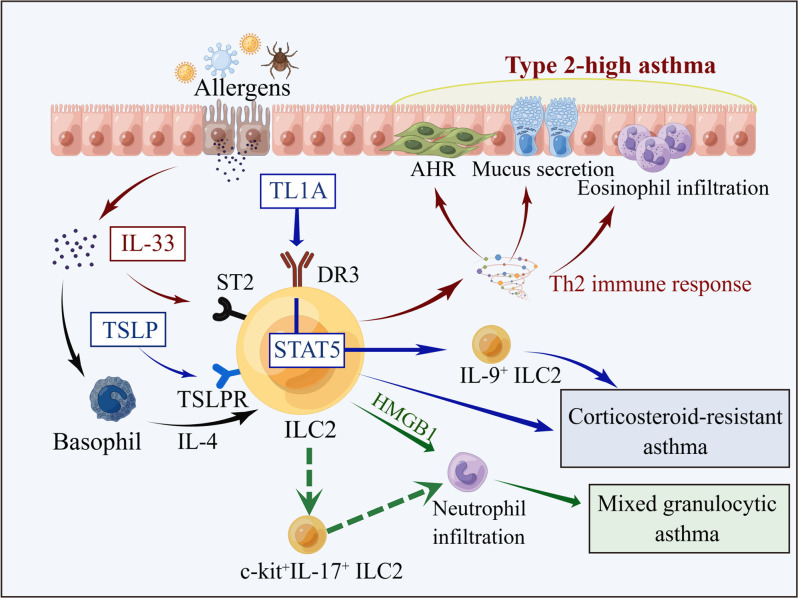
The IL-33-ILC2 axis drives type 2 airway inflammation in asthma. Upon epithelial injury, IL-33 activates ILC2s via ST2, inducing IL-5 and IL-13 secretion, which triggers eosinophilic inflammation, mucus hypersecretion, and airway hyperresponsiveness. Beyond canonical type 2 responses, ILC2s secrete high-mobility group box 1 (HMGB1) to recruit neutrophils and may acquire IL-17A-producing phenotypes under specific microenvironments, contributing to mixed granulocytic inflammation. thymic stromal lymphopoietin (TSLP) and TNF-like protein 1A (TL1A) synergize with IL-33 to induce corticosteroid resistance in ILC2s, while IL-33-induced basophil-derived IL-4 further enhances ILC2 activation in a positive feedback loop. Solid lines denote well−validated pathways; dashed lines denote hypothetical mechanisms that require further verification.

In summary, the IL-33-ILC2 axis in asthma serves as a core driver of type 2-high inflammation while simultaneously facilitating mixed granulocytic inflammation and steroid resistance through ILC2 plasticity and synergistic alarmin interactions. Translationally, these findings underscore the therapeutic imperative of targeting not only IL-33 itself but also the downstream effector functions and plasticity of ILC2s, particularly in patients with severe, steroid-resistant disease. The clinical efficacy of biologics such as dupilumab and benralizumab in severe asthma provides proof-of-concept for this approach (155–157) (**Figure 3**).

### COPD

COPD is a progressive lung disorder primarily caused by prolonged exposure to harmful particulate matter, particularly cigarette smoke. Traditional paradigms have held that COPD pathophysiology is driven primarily by type 1 and type 3 immunity, with type 2 cytokines and ILC2s playing only a limited role (158). However, accumulating clinical and experimental evidence has revealed substantial phenotypic heterogeneity, in which type 2 inflammation assumes a critical role in specific subtypes. Human evidence demonstrates patients with eosinophilic COPD (eCOPD) often exhibit elevated eosinophil counts in peripheral blood and airways, along with increased levels of type 2 cytokines such as IL-4 and IL-13, and show favorable responses to therapies targeting type 2 inflammatory pathways (159, 160). Moreover, virus‐triggered acute exacerbations are closely linked to ILC2-mediated type 2 immune responses in human patients, indicating that the IL-33-ILC2 axis exerts differential regulatory effects across disease states and clinical phenotypes (127).

A comparative examination of IL-33-ILC2 axis activation across different COPD phenotypes reveals both shared features and distinct divergences. Human evidence shows that in current smokers, persistent tobacco exposure leads to sustained IL-33 release from airway epithelium; however, the chronic inflammatory milieu, rich in IL-12 and IFN-γ, favors ILC2-to-ILC1 plasticity, ultimately resulting in a type 1‐dominant profile with reduced ILC2 numbers (24, 117). In former smokers, although baseline IL-33 levels decline after smoking cessation, residual structural damage and epithelial‐derived alarmins may sustain low‐grade chronic inflammation. Notably, a randomized clinical trial demonstrated that the anti-IL-33 monoclonal antibody itepekimab has demonstrated efficacy in reducing exacerbation rates and improving lung function in former smokers with COPD (161), suggesting that the IL-33 pathway remains therapeutically targetable even after cessation. Patients with asthma-COPD overlap (ACO) exhibit a mixed inflammatory signature, combining asthma‐related type 2 predominance with COPD‐typical airway remodelling and neutrophilic infiltration, and the response to glucocorticoids and biologic agents is highly heterogeneous (162–164). In eCOPD, type 2 inflammation is most pronounced; these patients often present with peripheral blood eosinophil counts ≥300 cells/μL, markedly elevated levels of IL-33, IL-5 and IL-13 in airway tissues, and greater ILC2 numbers and activation status (as indicated by Arg1 expression) than non-eosinophilic COPD patients. Moreover, the rate of lung function decline correlates positively with ILC2-derived cytokine concentrations (164–166). Thus, eCOPD likely represents the most sensitive subset for anti-IL-33/ILC2-targeted therapies (167, 168), and its acute exacerbation risk is closely associated with rapid ILC2 expansion triggered by IL-33.

The mechanistic basis underlying these phenotypic differences lies in the marked phenotypic plasticity of ILC2s, particularly their dynamic conversion toward ILC1s. It should be emphasized that current evidence for ILC2-to-ILC1 conversion is derived primarily from mouse models and *in vitro* human cell culture systems; direct *in vivo* evidence in human COPD lung tissue remains limited. In mouse models of cigarette smoke-induced COPD and *in vitro* human ILC2 cultures, IL-33 cooperates with signals such as IL-12 and IL-18 to downregulate the transcription factors Gata3 and Rora while upregulating T-bet, driving ILC2s to progressively lose type 2 cytokine production and acquire an ILC1-like phenotype capable of IFN-γ secretion (24, 117, 118, 120). In a seminal mouse study, Silver et al. (118) demonstrated that inflammatory triggers associated with COPD exacerbations orchestrate the plasticity of pulmonary ILC2s, driving their conversion to IFN-γ-producing ILC1-like cells. Human evidence corroborating this plasticity is indirect: in COPD patients, an inverse correlation between ILC2 and ILC1 frequencies in peripheral blood and lung tissue has been observed, and the ILC1/ILC2 ratio correlates with disease severity (24, 120). However, whether these circulating and tissue ILC1-like cells in COPD patients originate from ILC2 transdifferentiation or from *de novo* ILC1 differentiation from bone marrow progenitors has not been formally demonstrated in humans. Furthermore, whether the IFN-γ^+^ ILC1-like state observed in COPD represents a stable, committed lineage or a reversible adaptation to the chronic inflammatory milieu remains an unresolved question. Mouse lineage-tracing studies favor a model of dynamic, microenvironment-driven plasticity, but equivalent studies in human ILCs are lacking. During stable disease, particularly in severe COPD, human evidence demonstrates that pulmonary ILC2 populations contract while ILC1-like cells expand, reflecting persistent microenvironmental pressure (117, 120). In contrast, during virus-induced acute exacerbations, human evidence shows that serum IL-33 levels surge abruptly, rapidly activating and expanding residual ILC2s, accompanied by upregulation of Gata3 and Rora, which drive a burst of IL-5 and IL-13 release and consequently trigger severe type 2 airway inflammation (127, 169, 170). This dynamic switch suggests that ILC2 functional states are not fixed but are shaped by the prevailing cytokine environment; however, the precise molecular mechanisms governing this plasticity in the human lung require further elucidation.

Based on these mechanistic insights, the IL-33-ILC2 axis has emerged as a promising therapeutic target in COPD. Animal studies have confirmed that ILC2-specific deletion or functional inhibition (e.g., via Arg1 inhibitors) attenuates cigarette smoke-induced emphysema and airway inflammation (95, 171). Clinically, the anti-IL-33 antibody itepekimab has shown positive results in former smokers (161), while dupilumab (anti-IL-4Rα) provides clear benefits in eosinophilic COPD patients (159, 160). Translationally, given the heterogeneity of ILC2 activation status and plasticity propensities across phenotypes, future therapeutic strategies should be individualized according to phenotype and disease phase. For patients with eosinophilic COPD or those at high risk of exacerbations, early blockade of IL-33 or inhibition of ILC2 activation may be more effective; for those with severe stable disease dominated by type-1 immunity, combined modulation of the IL-12/IFN-γ pathway to limit excessive ILC1 polarization may be warranted. It is critical to recognize that in COPD, particularly in current smokers, IL-33-mediated effects are not exclusively ILC2-dependent. Chronic cigarette smoke exposure couples IL-33 release primarily to type 1 inflammation, and this effect is largely ILC2-independent. While IL-12 and IL-18 in the smoke-exposed lung drive ILC2s to convert into IFN-γ-producing ILC1-like cells (24, 117, 118), IL-33 also directly activates ST2-expressing CD8^+^ T cells and NK cells to produce IFN-γ, thereby amplifying type 1 immunity independently of ILC2 involvement (24). This dual mechanism—ILC2 plasticity plus direct activation of cytotoxic lymphocytes—explains the dominance of type 1 pathology in current smokers. Consequently, the elevated IL-33 levels observed in these patients do not reflect ILC2-driven type 2 inflammation but rather a broader IL-33-ST2-mediated type 1 response. These observations reinforce the importance of distinguishing ILC2-dependent from ILC2-independent IL-33 effects when interpreting biomarker data and designing targeted therapies for COPD.

**Figure 4:**
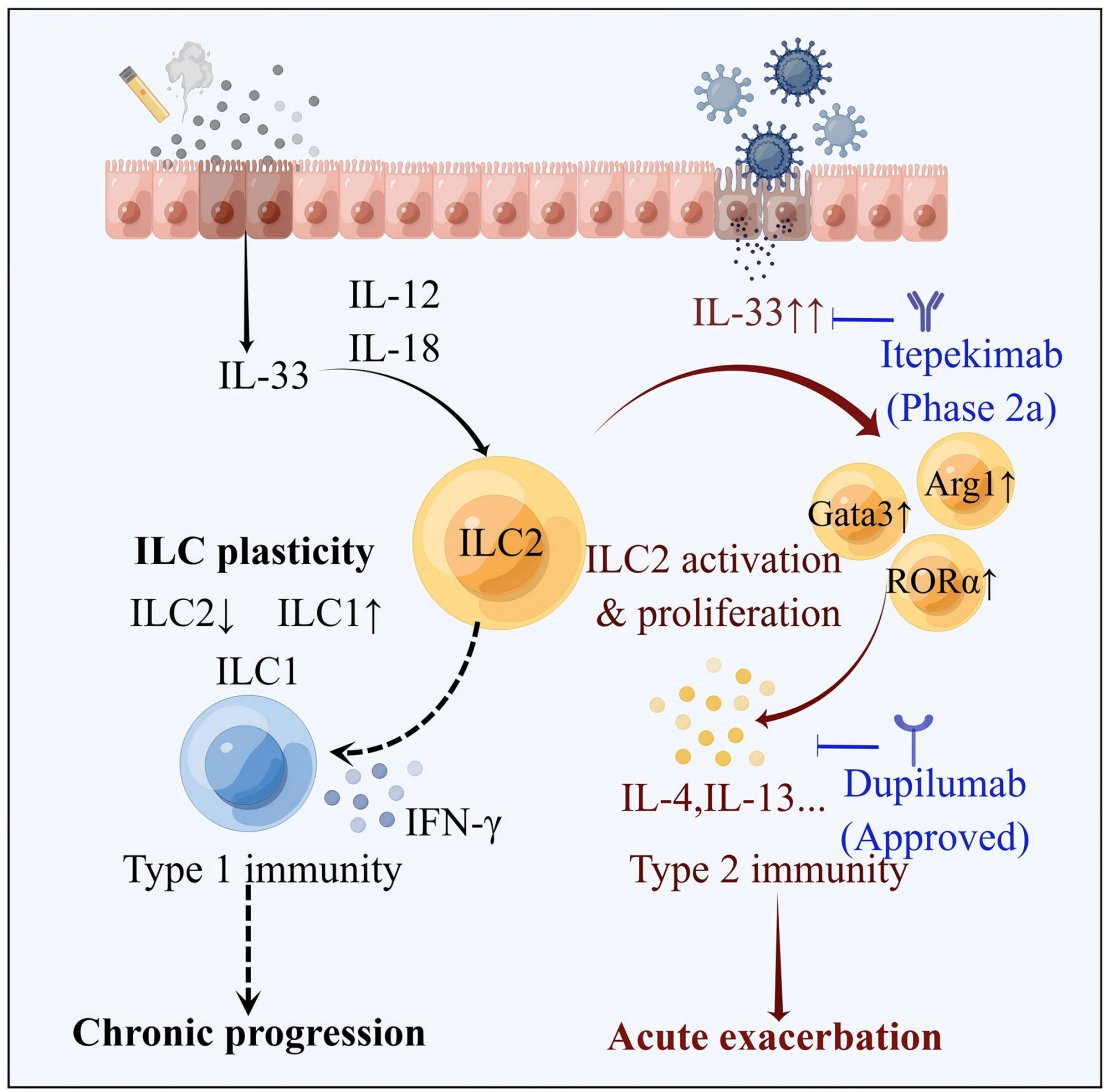
The IL-33-ILC2 axis drives divergent immune responses in stable versus acute exacerbation phases of COPD. During acute exacerbations (e.g., viral infection) in COPD, elevated IL-33 drives robust ILC2 activation and proliferation, accompanied by upregulation of Arg1, Gata3, and RORα, and triggers type 2 inflammation. This process is effectively blocked by itepekimab (anti–IL-33) and dupilumab (anti–IL-4Rα). In stable COPD, IL-12 converts ILC2s into IFN-γ-producing ILC1s, leading to ILC2 reduction and ILC1 expansion, which drives type 1 immunity and chronic disease progression (mouse/*in vitro* evidence; direct *in vivo* evidence in humans is limited). The dynamic ILC2/ILC1 imbalance underlies COPD heterogeneity. Solid lines denote well−validated pathways; dashed lines denote hypothetical mechanisms that require further verification.

Overall, the IL-33-ILC2 axis serves a dual role in COPD: its type-2 immune actions are key drivers of acute exacerbations, while its ILC2-to-ILC1 plasticity contributes to the maintenance of chronic type-1 inflammation. A deeper understanding of its differential regulation across phenotypes and disease phases will provide a theoretical foundation for precision-targeted therapies (**Figure 4**).

### Pulmonary fibrosis

Pulmonary fibrosis represents a heterogeneous pattern of lung remodelling rather than a single disease entity. Accumulating evidence indicates that the IL-33–ILC2 axis is a recurrent pro-fibrotic pathway in fibrotic lung disorders, but its function differs across idiopathic pulmonary fibrosis (IPF), cystic fibrosis (CF), systemic sclerosis-associated pulmonary fibrosis/interstitial lung disease (SSc-PF/SSc-ILD), and experimental bleomycin models. Human evidence shows elevated IL-33 levels in bronchoalveolar lavage fluid or sputum, together with increased ILC2 abundance or activation, have been reported in patients with IPF, CF, and related conditions (45, 93, 128, 172, 173). *In vitro*/mechanistic evidence indicates that IL-33 functions as an epithelial- or macrophage-derived alarmin that activates ST2-expressing ILC2s, promoting IL-13 production, M2 macrophage polarization, fibroblast activation, and extracellular matrix deposition (137, 174). However, the IL-33-ILC2 pathway should be interpreted as part of a broader IL-13/AREG/TGF-β regulatory network rather than as an isolated linear cascade.

In experimental bleomycin-induced pulmonary fibrosis, the IL-33–ILC2 axis has the strongest causal evidence. IL-33 is constitutively expressed in alveolar epithelial cells or induced in macrophages after lung injury (175). Inhibition of IL-33 signaling or depletion of relevant effector cells attenuates fibrosis, whereas recombinant IL-33 administration or adoptive transfer of ILC2s aggravates collagen deposition (175, 176). Hams et al. (45) further showed that ILC2s modulate fibrosis in anti-CD90.2 treated T and B cell deficient mice, and adoptive transfer experiments in *Il-13*^-/-^ mice demonstrated that IL-13-producing ILC2s directly regulate collagen accumulation. These findings define IL-13 as a principal ILC2-derived effector in the bleomycin model. Nevertheless, the model is highly dependent on the timing and intensity of epithelial injury, and therefore may preferentially capture an acute alarmin-driven type 2 response rather than the full chronicity of human IPF (177).

In IPF, the IL-33-ILC2 axis appears to act within a persistent epithelial injury–aberrant repair niche. Clinically, ILC2 frequency negatively correlates with the expression of the negative regulator Regnase-1, and increased ILC2 abundance predicts poor prognosis (178). At the mechanistic level, IL-33-induced activation of TGF-β promotes neuropilin-1 (Nrp1) expression, which enhances ST2 expression and further amplifies ILC2 function and type 2 immunity (179). Recent evidence also supports the relevance of the Nrp1/ILC2 pathway in IPF-related fibrosis, as targeting this axis attenuates experimental pulmonary fibrosis (122). In parallel, the AREG arm of the network is not restricted to ILC2s. Sustained AREG expression in intermediate alveolar stem cells drives progressive fibrosis in mouse fibrotic lungs and IPF patients, indicating that AREG may connect epithelial repair failure with fibroblast activation independently of, or in cooperation with, IL-33-activated ILC2s (129). *In vitro* evidence further shows that SULF1 promotes fibrosis through the TGF-β1/SMAD pathway in IPF, suggesting a feed-forward pro-fibrotic loop at the fibroblast level (180).

In CF, the IL-33-ILC2 pathway is also activated, but its role differs from that in IPF and bleomycin-induced fibrosis. CF is primarily driven by CFTR dysfunction, chronic epithelial stress, infection, and airway remodelling; therefore, IL-33-ILC2 activation likely acts as an amplifier of type 2 inflammation rather than as the initiating fibrotic mechanism. *In vitro* and *in vivo* evidence demonstrates that an IL-9-dependent positive feedback loop involving mast cells and Th9 cells enhances IL-25, IL-33, and ILC2 responses, whereas blockade of IL-9 or c-Kit (CD117) abrogates these effects (167). *In vitro* evidence using F508del CF airway epithelial cells shows that Akt-driven TGF-β and DKK1 secretion impairs epithelial polarity, linking epithelial dysfunction to aberrant remodelling (181). Thus, compared with IPF, CF-associated IL-33-ILC2 activity is more closely related to chronic epithelial inflammation and airway remodeling, whereas direct evidence for ILC2-driven interstitial collagen deposition is less established.

In SSc-PF/SSc-ILD, IL-33 and IL-13 appear to be markers and potential mediators of systemic immune-fibrotic activation. Human evidence demonstrates that serum IL-13 and IL-33 levels are increased in patients with SSc-ILD and correlate with impaired pulmonary function and radiological severity, supporting involvement of an IL-33/IL-13-skewed inflammatory milieu (123, 176, 182–184). However, compared with bleomycin-induced fibrosis and IPF, direct functional evidence that ILC2s are the dominant effector population in SSc-PF remains more limited. In this disease context, TGF-β signaling is a central downstream fibrotic hub; animal model evidence shows that nerandomilast improves bleomycin-induced SSc-ILD in mice by regulating the TGF-β1 pathway (185). Therefore, the IL-33-ILC2-IL-13 axis in SSc-PF is best understood as interacting with broader autoimmune and TGF-β-driven fibroblast activation pathways.

Taken together, the shared feature across these conditions is that IL-33 activates ILC2s and promotes type 2 effector signals, particularly IL-13, which can converge on macrophages, fibroblasts, and epithelial repair programs. The major differences lie in the disease-specific upstream triggers and downstream dominance of the network: bleomycin models emphasize acute epithelial injury and experimentally tractable IL-33–ILC2–IL-13 causality; IPF features chronic epithelial repair failure involving AREG and TGF-β-dependent fibroblast activation; CF links IL-33–ILC2 responses to IL-9-amplified airway inflammation and epithelial remodeling; and SSc-PF/SSc-ILD places IL-33/IL-13 within a systemic autoimmune-fibrotic and TGF-β-centered context. It is important to recognize, however, that IL-33 can promote pulmonary fibrosis through both ILC2-dependent and ILC2-independent mechanisms. While ILC2-derived IL-13 is a key fibrotic effector (45), IL-33 can also directly activate ST2-expressing fibroblasts and macrophages to produce TGF-β and extracellular matrix components, bypassing ILC2s entirely (45, 128). This dual mechanism—ILC2-mediated IL-13 production plus direct stromal cell activation—may explain why therapies targeting only one arm of the IL-33-ST2 network have shown variable efficacy across different fibrotic diseases, as exemplified by the limited effect of ST2-Fc in some experimental models (174).

**Figure 5:**
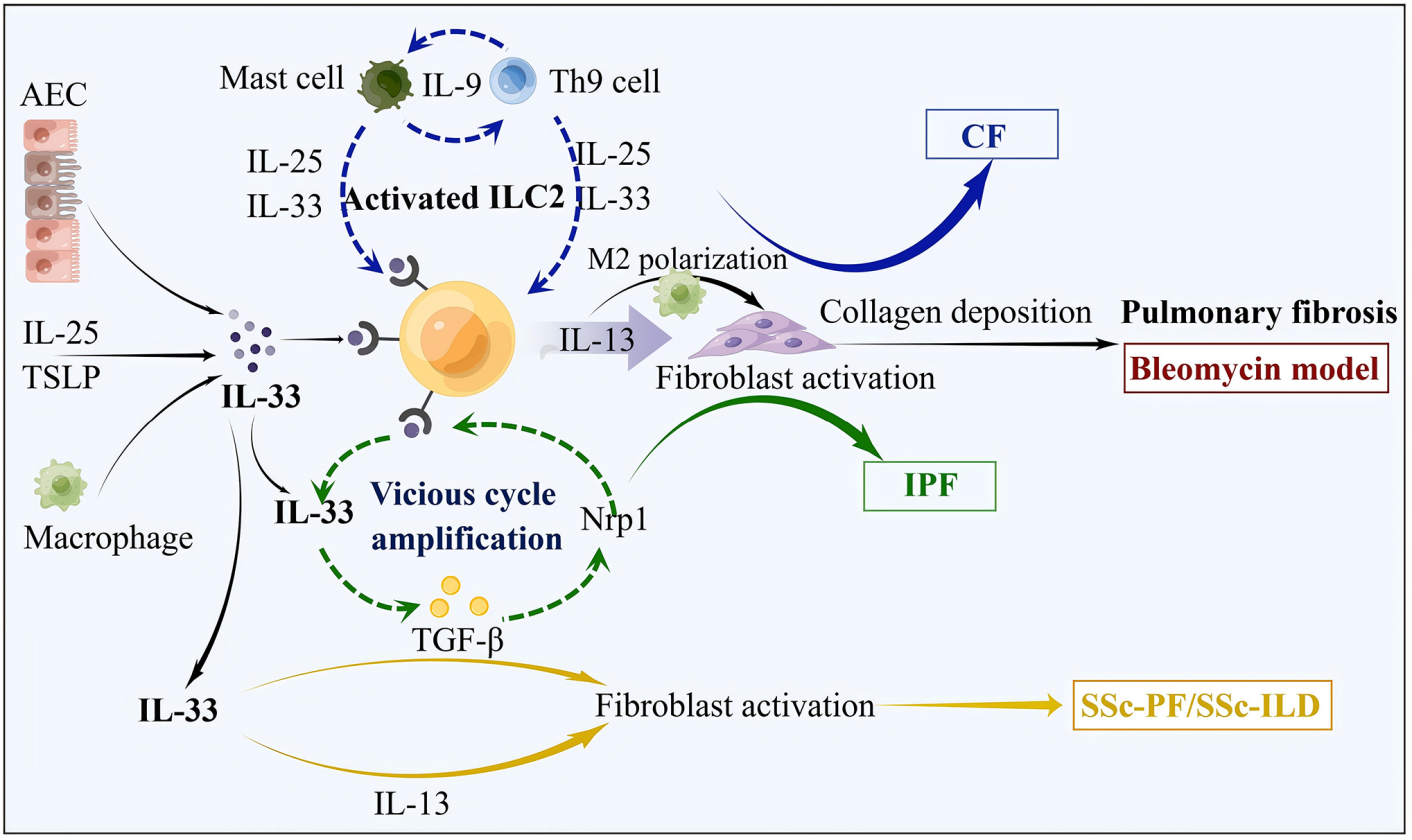
The IL−33−ILC2 axis drives pulmonary fibrosis through a multicellular network. Upon epithelial injury, alveolar epithelial cells (AECs) and macrophages release IL−33, which activates ILC2s to produce IL−13, promoting M2 macrophage polarization, fibroblast activation, and extracellular matrix deposition. Disease−specific features include: Bleomycin model – strongest causal evidence for the IL−33−ILC2−IL−13 axis; idiopathic pulmonary fibrosis (IPF) – IL−33 induces TGF−β/Nrp1−mediated ILC2 amplification, with AREG from intermediate alveolar stem cells contributing independently to fibrosis; cystic fibrosis (CF) – an IL−9−dependent positive feedback loop involving mast cells and Th9 cells amplifies ILC2 responses; systemic sclerosis-associated pulmonary fibrosis/interstitial lung disease (SSc-PF/SSc-ILD) – IL−33/IL−13 elevation occurs within a systemic autoimmune and TGF−β−driven context, with limited direct ILC2 evidence. Solid lines denote well−validated pathways; dashed lines denote hypothetical mechanisms that require further verification.

Translationally, although most studies support a pro-fibrotic role for this axis, IL-33–ST2 signaling may not be the sole or central driver in all settings. For example, Stephenson et al. (173) reported that therapeutic ST2-Fc did not identify IL-33-mediated ST2 signaling as a dominant fibrogenic pathway in bleomycin-induced pulmonary fibrosis. These findings suggest that therapeutic strategies targeting IL-33/ST2 or ILC2s should be tailored to disease context and may require combination with interventions directed at IL-13, AREG, or TGF-β-associated remodeling pathways (**Figure 5**).

### Viral respiratory infections

Respiratory virus-induced severe pulmonary diseases, which carry a significant global healthcare burden and are a major cause of morbidity and mortality, are strongly linked to robust type 2 immune responses. Across diverse viral infections, the IL-33/ILC2 axis plays complex yet pivotal roles, with its functional consequences ranging from protective tissue repair to pathological inflammation, depending on the viral strain and host context (123, 124, 182–184, 186).

During influenza virus infection, the IL-33-ILC2 axis displays a unique dynamic duality in mouse models. Viral infection activates the NLRP3 inflammasome in alveolar macrophages and NKT cells, triggering IL-33 release that subsequently drives ILC2s to produce IL-13 and IL-5. This induces type 2 inflammatory pathology, including airway hyperresponsiveness and eosinophil recruitment (187). In animal studies, this process is tightly regulated by type I interferons and IFN-γ via STAT1-dependent pathways. In the absence of these inhibitory signals, IL-33-mediated ILC2 activation is significantly enhanced (123). Importantly, as viral loads decline in the late stages of infection, the function of ILC2s shifts toward a protective role: they produce AREG, which promotes epithelial repair, and leukemia inhibitory factor (LIF), which promotes immune cell migration away from the lungs, thereby limiting chronic inflammation (188). In animal and *in vitro* studies, this functional transition from pathogenic inflammation to protective repair is regulated by the AP-1 transcription factor BATF and suggests the potential existence of functionally distinct ILC2 subpopulations (189), highlighting the complex mechanisms that balance immune defense and tissue homeostasis.

In contrast to influenza, during respiratory syncytial virus (RSV) infection, the IL-33-ILC2 axis primarily drives harmful immunopathological responses, which is a characteristic that is particularly prominent in infancy and early childhood. Upon infecting airway epithelial cells, RSV induces the release of alarmins, including IL-33, IL-25, and TSLP, with TSLP playing a more critical driving role in RSV infection than in other viral infections (190, 191). TSLP synergizes with IL-33 to activate ILC2s, promoting substantial secretion of IL-13 and IL-5, which leads to characteristic pathological changes, including airway hyperresponsiveness, excessive mucus secretion, and eosinophilia. In animal studies, this aberrant ILC2 response is strongly associated with an inadequate host type 1 immune response; insufficient or delayed IFN-γ production during infancy and early childhood weakens its direct inhibitory effect on ILC2s (192–194). In human and animal evidence, this pathological response exhibits notable age and sex differences. Neonates (particularly males) have higher levels of ILC2s and sustained activation that persists for up to 4 weeks. This correlates with weaker Th1 responses and the incomplete regulatory effects of testosterone prior to puberty, collectively increasing the risk of long-term asthma following early-life RSV infection (195–198).

During early rhinovirus (RV) infection, IL-33 also forms a critical network with IL-25 and TSLP to activate ILC2s in mouse models (199–202). Similar to RSV infection, neonatal RV infection significantly increases the number of IL-13-producing ILC2s in the lungs, yet its initiation mechanism differs substantially. IL-25 is the critical initial trigger, whereas IL-33 and TSLP serve as essential synergistic signals for full ILC2 activation (183, 199). In mouse models, a proposed mechanism underlying this pathological response in infancy and early childhood involves the absence or delayed development of Th1/IFN-γ immunity. Experimental evidence shows that supplementation with IFN-γ or pharmacological blockade of the key ILC2 transcription factor ROR-α using either the ROR-α inhibitor SR3335 or via *ROR-α* gene knockout (complete *ROR-α* knockout mice or conditional knockout mice specifically targeting ILC2s) reduces disease severity (200–202). In this context, the IL-33-ILC2 axis is predominantly pathogenic, with no clearly established protective repair phase.

In SARS-CoV-2 infection, the IL-33-ILC2 axis is dynamically linked to disease severity through antagonism with type I and type II interferons. Critically ill patients, especially the elderly, exhibit elevated systemic IL-33 (203), while circulating ILC2 numbers are higher in moderate disease and decrease in severe cases (204).This pattern directly reflects the intensity of the IFN-I/IFN-γ response: in a high-IFN-γ environment: ILC2s are suppressed by high IFN-γ, but can promote AREG-mediated tissue repair when interferon signaling wanes (182, 188). Compared with influenza, COVID-19 outcomes are more variable and closely tied to the cytokine microenvironment and interferon dysregulation. Thus, the IL-33-ILC2 axis in COVID-19 represents an interferon-dominated tug-of-war, highlighting a therapeutic target shared with yet distinct from that in other respiratory viruses.

A concise comparison reveals both convergent and divergent features of the IL-33-ILC2 axis among these four viruses. A shared mechanism lies in the virus-induced release of IL-33 from airway epithelial cells, which couples with other alarmins (TSLP, IL-25) to activate ILC2s. In all infections, the resulting ILC2 response is negatively regulated by type I and type II interferons, explaining the heightened susceptibility in early life when IFN responses are immature. However, distinct features differentiate the pathological outcomes. The dominant alarmin driving ILC2 activation varies: TSLP plays a more prominent role in RSV infection, whereas IL-25 is the critical initiator for RV. The functional outcome of ILC2 activation also differs markedly. Influenza uniquely exhibits a clear, BATF-dependent transition from early IL-5/IL-13-mediated immunopathology to late AREG/LIF-mediated tissue repair. In contrast, RSV and RV infections in early life are characterized by a sustained, pathogenic ILC2 response with minimal functional reversion toward repair. SARS-CoV-2 sits in a more complex position, where the balance between pathology and repair is not temporally programmed but is instead dynamically determined by the degree of interferon dysregulation, leading to highly variable outcomes. This comparative framework underscores that therapeutic strategies targeting the IL-33-ILC2 axis must be tailored to the specific viral context and host age. In viral infections, IL-33 also exerts ILC2-independent effects by directly activating CD8^+^ T cells and NK cells to produce IFN-γ, thereby promoting antiviral type 1 immunity (24). This adds complexity to the dual role of the IL-33-ILC2 axis and suggests that complete therapeutic blockade of IL-33 could inadvertently impair beneficial antiviral responses while suppressing type 2 immunopathology.

**Figure 6:**
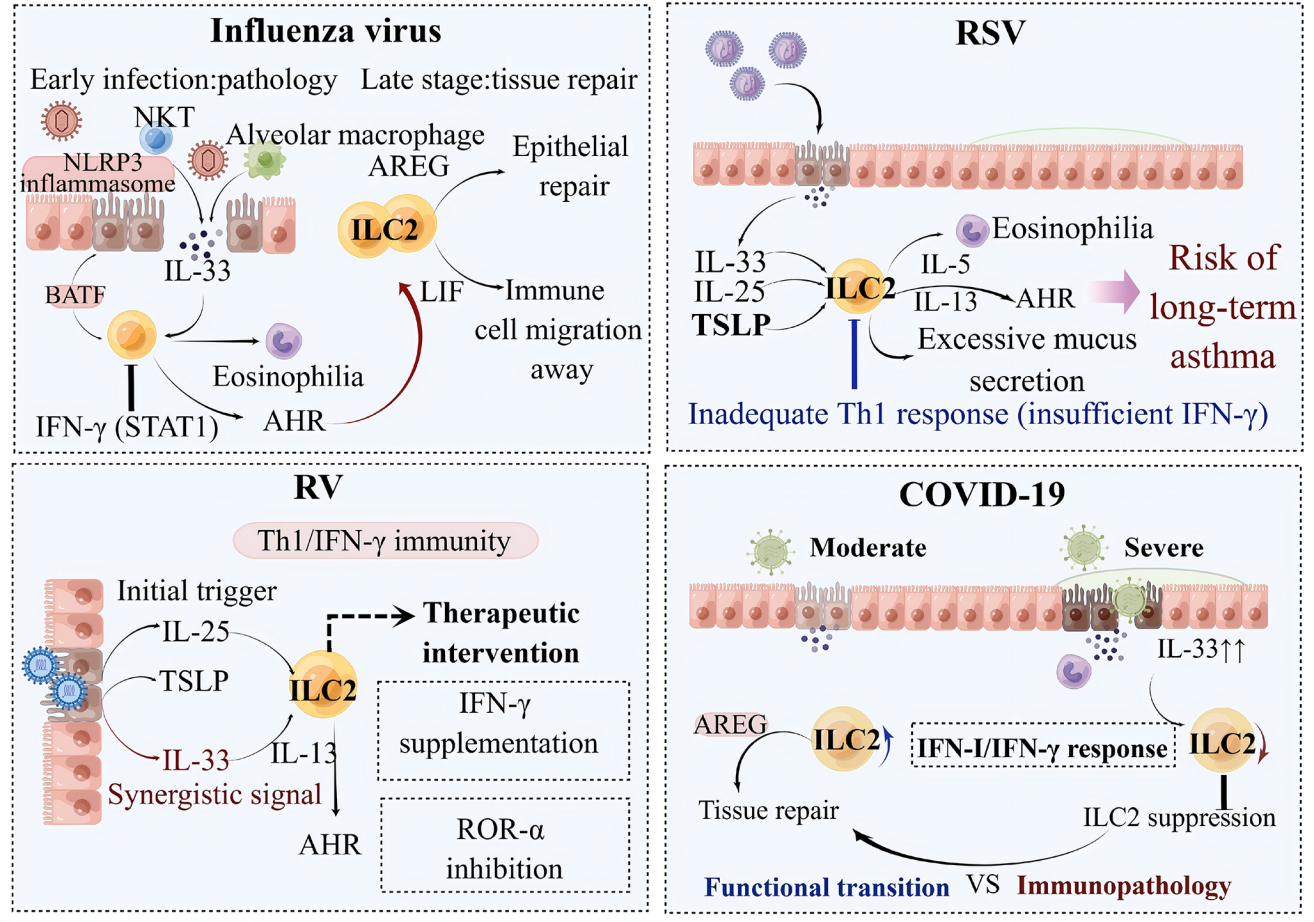
The IL-33-ILC2 axis exhibits virus-specific and context-dependent functions in respiratory viral infections. In influenza, IL−33 drives early ILC2−mediated type 2 pathology (IL−5/IL−13), followed by a BATF−regulated transition to protective AREG/LIF−mediated tissue repair. In RSV infection, IL-33 synergizes with TSLP to activate ILC2s, leading to airway hyperresponsiveness (AHR), mucus hypersecretion, and eosinophilia, particularly in infants with inadequate IFN-γ responses, increasing long-term asthma risk. In RV infection, IL-25 serves as the initial trigger, with IL-33 and TSLP as essential co-signals for full ILC2 activation; this process is suppressed by IFN-γ or ROR-α inhibition. In SARS−CoV−2, the axis operates in an interferon−dominated balance, where ILC2s are suppressed by high IFN−γ but may support repair when interferon signaling wanes. Solid lines denote well−validated pathways; dashed lines denote hypothetical mechanisms that require further verification.

In summary, IL-33 serves as a pivotal bridge linking viral infection to type 2 immunity, while ILC2s, as effector cells, exhibit highly context-dependent functions: driving immunopathology in RSV and RV, performing a dual role in influenza, and engaging in an interferon-dependent push-pull in SARS-CoV-2. Targeting the IL-33-ILC2 axis or modulating the balance between type 1 and type 2 immunity holds promise as an innovative therapeutic strategy for specific viral respiratory diseases (**Figure 6**).

## Therapeutic prospects and challenges of targeting the IL-33-ILC2 axis

The studies discussed in this review collectively establish the IL-33-ILC2 axis as a central regulatory hub in respiratory diseases, making it an attractive therapeutic target. Current strategies are organized along three levels: upstream blockade of IL-33/ST2 signaling, neutralization of ILC2-derived effector cytokines, and indirect modulation of ILC2 function (**Table 3**). However, translating these approaches into clinical practice faces several fundamental challenges.

**Table 3:** Therapeutic strategies targeting the IL-33-ILC2 axis in respiratory diseases.

Category	Agent	Target	Indication (s)	Clinical stage	Main findings	Limitations	Refs
Direct blockade of IL-33/ST2	Itepekimab	IL-33	COPD	Phase 2a	Reduced exacerbation rate and improved FEV_1_ in former smokers; no benefit in current smokers	Benefit restricted to former smokers; no effect in current smokers; larger confirmatory trials needed	(161)
	Etokimab (ANB020)	IL-33	Severe eosinophilic asthma	Phase 2a	Rapid and sustained improvement in FEV1 and ACQ-5 score reduction.; eosinophil reduction	Limited evidence from small trial	(205)
	Astegolimab	ST2^*^	Severe uncontrolled asthma	Phase 2b (ZENYATTA)	490 mg dose significantly reduced annualized exacerbation rate; greater benefit in eosinophil-high subgroup	Only the 490 mg dose effective; FEV_1_ not significantly improved	(206)
	CNTO7160	ST2	Asthma	Phase 1	Safety and tolerability established; pharmacokinetics characterized	Efficacy not established; clinical development status unclear	(207)
	Torudokimab (LY3375880)	IL-33	Not yet defined	Preclinical	High-affinity IL-33 neutralization; inhibited IL-33-dependent signaling *in vitro*	Not tested in humans; therapeutic potential unknown	(208)
Targeting ILC2 effector cytokines	Dupilumab	IL-4Rα (blocks IL-4/IL-13)	Severe eosinophilic asthma; COPD with type 2 inflammation	Approved	Reduced exacerbations, improved lung function in eligible patients	Efficacy limited in non-eosinophilic or mixed granulocytic phenotypes; potential impact on tissue repair unclear	(159, 160)
	Mepolizumab	IL-5	Severe eosinophilic asthma	Approved	Reduced exacerbations and oral corticosteroid use; recent evidence suggests attenuation of airway remodeling	Only effective in eosinophilic phenotype; impact on long-term disease modification requires further study	(209)
	Benralizumab	IL-5Rα	Severe eosinophilic asthma	Clinical study (single-blind, placebo-controlled)	Reduced exacerbations; normalized sputum eosinophilia even after anti-IL-5 failure	Depletes eosinophils and basophils; limited efficacy in non-eosinophilic inflammation; may spare ILC2s	(157)
Indirect modulation of ILC2 function	R848 (TLR7/8 agonist)	TLR7/8 (macrophages)	Severe asthma (exploratory)	Preclinical (animal models)	Suppressed ILC2-mediated airway inflammation via IL-27 induction from macrophages	No human data; potential for off-target immune activation; dosing and delivery route undefined	(210)
	Long-acting muscarinic antagonists (e.g., tiotropium)	Muscarinic receptors (basophil-dependent pathway)	COPD, asthma	Approved (for bronchodilation); ILC2-modulating effect shown in preclinical models	Attenuated ILC2-dependent eosinophilic airway inflammation in mice; proposed mechanism involves basophil modulation	ILC2 inhibition is an off-target effect; clinical relevance for ILC2 modulation in humans not yet demonstrated; precise mechanism remains unclear	(211)

### Blockade of the IL-33/ST2

Several monoclonal antibodies targeting IL-33 or its receptor ST2 have entered clinical development, with mixed results. For IL-33 blockade, itepekimab (anti-IL-33) demonstrated efficacy in a phase 2a trial in former smokers with moderate-to-severe COPD, reducing exacerbation rates and improving lung function, but this benefit was not observed in current smokers, suggesting that the therapeutic window for anti-IL-33 therapy in COPD is restricted by smoking status (161). Etokimab (ANB020), another anti-IL-33 antibody, showed preliminary evidence of improved lung function in a phase 2a trial in severe eosinophilic asthma, although the primary endpoint was not met, and larger confirmatory studies are awaited (205). Astegolimab (anti-ST2) significantly reduced the annualized asthma exacerbation rate at the 490 mg dose in the phase 2b ZENYATTA trial in severe uncontrolled asthma (RR 0.70, p=0.018), with a more pronounced effect in the subgroup with blood eosinophils ≥300 cells/μL (206). CNTO7160, an anti-ST2 monoclonal antibody, was evaluated in a first-in-human study in healthy subjects and patients with asthma or atopic dermatitis, but its clinical development status remains unclear (207). Torudokimab (LY3375880), a high-affinity anti-IL-33 antibody, remains in preclinical development (208). These mixed results illustrate that IL-33/ST2 blockade is not uniformly effective and that patient stratification based on phenotype, disease phase, and smoking history is likely essential for clinical success.

### Targeting ILC2 effector cytokines

Therapies targeting ILC2-derived cytokines have shown more consistent clinical benefits, particularly in type 2-high patient populations. Dupilumab (anti-IL-4Rα), which blocks both IL-4 and IL-13 signaling, has demonstrated robust efficacy in severe eosinophilic asthma and in COPD patients with elevated blood eosinophil counts (159, 160). Mepolizumab (anti-IL-5) reduces exacerbations and oral corticosteroid use in severe eosinophilic asthma, and recent evidence suggests that it may also attenuate airway remodeling, as demonstrated by reductions in airway wall thickness on HRCT and decreases in remodeling-associated mediators such as TGF-β1 and MMP-9 in patients with late-onset severe eosinophilic asthma (209). This finding supports the concept that targeting ILC2-derived cytokines may not only control inflammation but also modify disease progression. Benralizumab (anti-IL-5Rα) has proven effective in reducing exacerbations and normalizing sputum eosinophilia even in patients with inadequate response to anti-IL-5 therapy (157). However, these agents primarily address the downstream consequences of ILC2 activation rather than ILC2s themselves, and their efficacy is largely confined to patients with an eosinophilic phenotype.

### Indirect regulatory strategies

Beyond direct blockade, indirect approaches to modulate ILC2 function are being explored preclinically. The TLR7/8 agonist R848 suppressed ILC2-mediated inflammation by inducing IL-27 production from interstitial macrophages (210), while long-acting muscarinic antagonists attenuated ILC2-dependent airway eosinophilia through basophil-mediated pathways (211). These findings suggest that pharmacological reprogramming of the local microenvironment may offer an alternative to direct cytokine blockade, though clinical validation is lacking.

Despite this progress, three key challenges remain. First, would complete IL-33 blockade impair tissue repair? The dual role of the IL-33-ILC2 axis—driving both pathogenic inflammation and protective repair—raises this concern. ILC2s produce AREG and LIF during late-phase influenza, facilitating epithelial repair and resolving chronic inflammation (188, 189). In the neonatal lung, an AREG-expressing reparative ILC2 subset supports normal alveolar development (136). Sustained IL-33 blockade could theoretically compromise these repair processes, particularly during ongoing tissue injury. Thus, therapeutic timing and dosing must be carefully considered: in influenza, early blockade may avert immunopathology, whereas late blockade could impair AREG-mediated restitution; in BPD, restoring the balance between pro-inflammatory and reparative ILC2 subsets may be preferable to complete axis suppression.

Second, how can pathogenic and reparative ILC2s be distinguished for selective targeting? Single-cell analyses have begun to define their molecular signatures. Pro-inflammatory ILC2s express Il5, Il13, Arg1, and Klrg1, whereas reparative ILC2s express Areg and Icos (136). Functionally, the transcription factor BATF orchestrates the transition from pathogenic to reparative ILC2s during influenza (189), suggesting that this transcriptional switch could be therapeutically modulated. Surface markers such as ST2, KLRG1, ICOS, and PD-1 may enable antibody-mediated depletion of pathogenic ILC2s while sparing their reparative counterparts. However, this approach remains entirely preclinical, and whether these markers faithfully segregate function in human disease or ILC2s reversibly transition between states depending on microenvironmental cues is unknown.

Third, is targeting ILC2 plasticity in COPD feasible? In current smokers, IL-12 and IL-18 drive the conversion of ILC2s into IFN-γ-producing ILC1-like cells (117, 118), a process that correlates with disease severity (120, 164). The differential efficacy of itepekimab in former versus current smokers (161) provides proof-of-principle that this conversion is at least partially reversible upon smoking cessation. Pharmacological blockade of IL-12 or IL-18, or transcriptional stabilization of ILC2 identity by enhancing GATA3 or inhibiting T-bet, could theoretically preserve a targetable type 2 axis even in current smokers. However, ILC2 plasticity is deeply embedded in the chronic inflammatory milieu of the smoker’s lung, and blocking ILC1 conversion might impair anti-viral and anti-tumor type 1 responses. Trials combining smoking cessation with anti-IL-33 or anti-IL-12/IL-18 therapies would be informative but have not yet been initiated.

In summary, the therapeutic promise of the IL-33-ILC2 axis is tempered by the functional duality and plasticity of ILC2s. Progress will require biomarkers that distinguish pathogenic from reparative responses, therapeutic regimens timed to disease phase and tailored to phenotype, and clinical trials that account for the dynamic nature of ILC2 function.

## Conclusion and future perspectives

The IL-33-ILC2 axis functions as a central regulatory hub of airway type 2 immunity, yet its role extends well beyond the traditional paradigm of eosinophilic inflammation (1). Across the respiratory diseases reviewed here, the functional output of this axis is shaped by the local microenvironment, leading to disease-specific patterns of ILC2 activation, plasticity, and effector function. In asthma, the axis drives type 2-high eosinophilic inflammation (126), while also contributing to mixed granulocytic inflammation through ILC2 plasticity and HMGB1 secretion (119, 150) and to steroid resistance via alarmin synergy (121, 153). In COPD, the axis is governed by a dynamic equilibrium between ILC2-driven type 2 responses and ILC2-to-ILC1 plasticity, the balance of which is dictated by smoking status and determines therapeutic responsiveness (24, 118). In BPD, the physiological role of the axis in supporting normal alveolar development is subverted by hyperoxia, which drives overactivation of pro-inflammatory ILC2s and arrests alveolarization (136, 137). In pulmonary fibrosis, a conserved IL-33-ILC2-IL-13/AREG/TGF-β network drives fibroblast activation (45, 173), but its contribution varies from a dominant causal role in experimental models to an amplifier function in human IPF, CF, and SSc-ILD. In viral infections, the axis exhibits a striking functional duality, mediating immunopathology in RSV and RV (190, 199), performing a temporally programmed dual role in influenza (187, 189), and participating in an interferon-dependent tug-of-war in SARS-CoV-2 (203, 204).

It must be acknowledged that the current understanding of ILC2 plasticity—particularly the conversion into ILC1-like or IL-17-producing subsets—is derived largely from mouse models and *in vitro* induction systems. Direct evidence for such plasticity in human respiratory diseases is emerging but remains limited, with key studies constrained by small sample sizes and the technical challenges of definitively tracing ILC2 lineage in human tissues. Furthermore, a fundamental question remains unresolved: whether the distinct functional states observed (e.g., IFN-γ^+^ ILC2s, IL-17A^+^ ILC2s) represent stable, developmentally committed subsets or dynamic, reversible adaptations to the local cytokine milieu. Current evidence from mouse lineage-tracing and adoptive transfer experiments favors the dynamic adaptation model, but definitive proof in the human lung is lacking. In asthma, ILC2s can drive typical type 2 inflammation, and human evidence suggests that specific ILC2 subsets may contribute to neutrophilic inflammation and mixed granulocytic responses via IL-17 secretion (117, 119), though these findings require further validation in larger cohorts. In COPD and pulmonary fibrosis, evidence from mouse models and *in vitro* studies indicates that cytokines such as IL-12 and IL-18 can promote switching of ILC2s to an ILC1-like phenotype, thereby contributing to type 1 inflammation and tissue remodelling (24, 117, 118). Conversely, during viral infections, mouse studies show that ILC2s exhibit a dual role, mediating immunopathology while simultaneously supporting tissue repair through AREG production (123, 124). This extensive functional heterogeneity highlights the multifaceted role of ILC2s in airway diseases, but its relevance to human pathophysiology will require more rigorous validation using advanced techniques such as single-cell multi-omics and lineage-tracing in human tissue samples.

Importantly, these disease-specific functions must be understood within the broader context of the IL-33-ST2 signaling network. IL-33 acts on a diverse array of ST2-expressing cells—including Tregs, mast cells, basophils, eosinophils, macrophages, NK cells, Th cells, and CD8^+^ T cells—and the net pathological outcome depends on which cell types predominate in a given disease context. In eosinophilic asthma, ILC2s are the dominant IL-33 targets, and the axis drives canonical type 2 inflammation. In current smokers with COPD, by contrast, IL-33 primarily activates CD8^+^ T cells and NK cells, eliciting type 1 responses that are largely ILC2-independent (24). In viral infections, the interplay between IL-33-mediated ILC2 activation and direct IL-33-driven antiviral type 1 immunity shapes the functional duality of ILC2s (123, 182). Recognizing this complexity—and distinguishing ILC2-dependent from ILC2-independent IL-33 effects—is essential for the rational design of therapies that target the appropriate arm of the IL-33-ST2 network in each disease.

Looking ahead, three priorities will shape the translation of these insights. First, single-cell multi-omics technologies should be harnessed to define the molecular signatures of pathogenic versus reparative ILC2 subsets in human disease, enabling the development of biomarkers for patient stratification and selective targeting. Second, clinical trials of IL-33/ST2-directed therapies must incorporate phenotypic and endotypic stratification to identify the patient subsets most likely to benefit, as illustrated by the differential efficacy of itepekimab in former versus current smokers. Future research should focus on guiding patient stratification through disease-specific ILC2 biomarkers and developing personalized therapies targeting distinct pathogenic subpopulations. Third, a deeper understanding of the transcriptional and epigenetic mechanisms governing ILC2 plasticity—including the GATA3/T-bet balance and BATF-mediated functional switching—may reveal novel therapeutic nodes for modulating ILC2 function without global immunosuppression. By addressing these challenges, the field can move toward a precision immunotherapy paradigm in which the IL-33-ILC2 axis is not simply blocked, but rather therapeutically tuned to restore immune homeostasis across the spectrum of respiratory diseases.
